# Polymeric micelles in advanced photodynamic therapy: Design, delivery and translational prospects

**DOI:** 10.1016/j.ijpx.2025.100439

**Published:** 2025-11-06

**Authors:** Alžběta Turnovská, Tomáš Etrych

**Affiliations:** Institute of Macromolecular Chemistry, Czech Academy of Sciences, Heyrovského nám. 2, Prague 6, 162 00, Czechia

**Keywords:** Polymer, Micelle, Release, Encapsulation, Conjugation

## Abstract

Photodynamic therapy (PDT) is widely studied and complex method useful as a minimally invasive cancer treatment strategy, relying on photosensitizers (PSs), light, and oxygen to induce cytotoxicity. Indeed, the controlled delivery of conventional PSs is the key factor in achieving effective treatment outcome. Among many drug delivery systems, the polymeric micelles represent a promising platform to address the solubility, stability, and delivery challenges associated with PSs. The design of micelles, constructed from hydrophilic and hydrophobic polymeric blocks in diverse structures, enables precise tailoring of carrier properties to optimize PS delivery. This paper focuses on the potential applications and limitations of polymer micelles for the controlled delivery of PSs in the field anticancer therapy. Various methods of synthesis, incorporation of PSs as well as their release and activation are described in detail. The effect of micellar system employment on circulation time, off-target effects, and both passive and active targeting are thoroughly depicted. Despite the clinical promise, the limitations of PDT including shallow tissue penetration and restricted applicability to superficial or endoscope-accessible tumors are discussed, as well as the future prospects consisting in red-shifted or two-photon absorption systems.

## Highlights

• Provides in-depth overview of polymer micelles used in photodynamic therapy (PDT).
• Summarizes commonly used materials and structures relevant to carrier design.
• Discusses both physical encapsulation and chemical conjugation of photosensitizer.
• Explores various external/internal stimuli for controlled release.
• Highlights advantages, future challenges and directions for micelles in PDT.

## Graphical abstract

**Figure:**
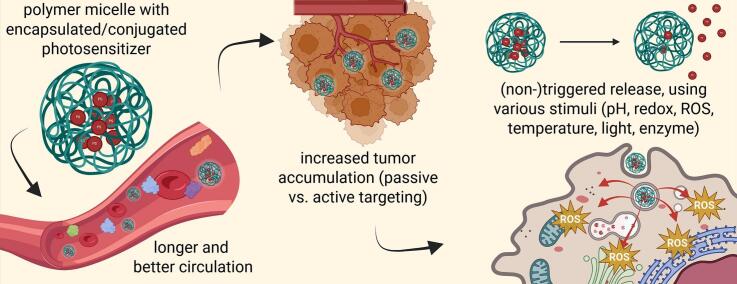

## 1 Introduction

Photodynamic therapy (PDT) is a minimally invasive treatment modality employed to treat various malignant tumors. It is a clinically used procedure that has gained attention over conventional therapies, such as chemotherapy, radiotherapy, or surgery. Several drugs were approved by regulatory authorities for bladder and esophageal cancer treatment in 1993 and 1994, respectively (Penetra et al., 2023). Since then, PDT has found entered clinical trials for head and neck (Ramsay et al., 2021; van Doeveren et al., 2018), lung (Allison and Bansal, 2022; Kato et al., 2003), skin (De Rosa and Bentley, 2000; Kochneva et al., 2010) or prostate (Moore et al., 2006; Nathan et al., 2002) cancer.

PDT relies on the energy transfer between photosensitizer (PS), which is excited into higher electronic states by illumination with an appropriate wavelength, and molecular oxygen presented in the tumorous tissue. (Kübler, 2005) Upon illumination, the PS absorbs energy and is excited from its ground state (S0) to higher electronic states (S1, S2, …). From there, several radiative or non-radiative processes (i.e. internal conversion, vibrational relaxation, fluorescence, intecrsystem crossing, phosphoresnce) can occur. Excited PS* in its longer-lived triplet state can participate in other photochemical processes and reactions. (Dąbrowski, 2017; Plaetzer et al., 2009).

Two types of photochemical reaction involving the excited PS^⁎^ are known, Type I and Type II. In a Type I reaction, the PS^⁎^ transfers an electron or proton oto a substrate, such as cell membranes or surrounding molecules, initiating a cascade of so-called reactive oxygen species (ROS) production, including superoxide ion O_2_^•-^, perhydroxyl radicals HO_2_^•-^, hydrogen peroxide H_2_O_2_, or hydroxyl radical HO^•^. (Correia et al., 2021a; Dabrowski, 2017; Kwiatkowski et al., 2018) In a Type II reactions, the excited PS* in its triplet state interacts directly with molecular oxygen, yielding two singlet oxygen ^1^O_2_ forms (^1^Σ_2_, ^1^Δ_2_). Due to very short life time of O_2_(^1^Σ_2_) it is predominantly the lower-energy form O_2_(^1^Δ_2_) that is relevant to PDT. (Dabrowski, 2017; Josefsen and Boyle, 2008a) All of these highly reactive ROS then create oxidative stress by oxidizing proteins, carbohydrates or lipids, and initiate biological cascades, ultimately leading to tumor cell apoptosis or necrosis. Moreover, PDT can destroy tumor's vasculature and essentially deprive it of nutritients, or stimulate tumor cells to release damage-associated molecular patterns (DAMPs), hence activating the immune system. The latter constitutes a major benefit over conventionally used therapies, as they are usually immunosuppressive (Donohoe et al., 2019). (Abels, 2004; Castano et al., 2005a; Nowis et al., 2005).

Photosensitizers play a crucial role in PDT, since they are responsible for light absorption and reactions with molecular oxygen and surrounding molecules. Ideally, they should preferentially accumulate inside the tumorous tissue, without affecting healthy cells. For successful PDT treatment, they should also possess low activity in the absence of light and exert high triplet state and ^1^O_2_ quantum yields upon illumination. Over the course of time, three generations of photosensitizers have been distinguished, with each generation overcoming the limitations of their predecessors. (Correia et al., 2021a; Lucky et al., 2015).

The first generation included porphyrin-based PS such as hematoporphyrin derivative (HpD) or porfimer sodium. Despite their low dark cytotoxicity, they also accumulated in the healthy tissues, causing prolonged photosensitivity and requiring high doses for sufficient PDT effect due to their low absorption coefficients. The seconds generation PSs, based on chlorins, bacteriochlorins, phthalocyanines, or porphyrins offered stronger absorption, higher ^1^O_2_ quantum yields, and reduced photosensitivity, but their poor solubility and low hydrophilicity still limited their intravenous administration. These drawbacks led to the development of third-generation PSs, which employ delivery vehicles or carriers, such as liposomes, amino acids, peptides, polymeric micelles, two-dimentional (2D) nanomaterials and many more, to improve solubility, tumor selectivity and overall therapeutic efficacy. (Correia et al., 2021a; Le Clainche et al., 2025; Lucky et al., 2015; Luo et al., 2024; Mfouo-Tynga et al., 2021; Xu et al., 2025; Yu et al., 2022).

Polymeric micelles represent a particularly advantageous class of nanocarriers for PSs´ delivery. Formed by the self-assembly of amphiphilic copolymers, they possess a hydrophobic core that can efficiently encapsulate poorly soluble PSs and a hydrophilic corona that enhances aqueous stability, biocompatibility and prolongs systemic circulation. (Matsumura and Maeda, 1986) These structural features not only improve the solubility and bioavailability of hydrophobic PSs but also promote passive tumor accumulation through the enhanced permeability and retention (EPR) effect. Moreover, polymeric micelles can be engineered with stimuli-responsive or targeting moieties to enable controlled release and selective delivery, thereby reducing off-target toxicity. Compared with other delivery platforms, micelles offer simple preparation, tunable properties, and favorable biocompatibility, making them highly suitable for third-generation PS design. (Chen et al., 2020; Liu et al., 2020; Potara et al., 2021; Shieh et al., 2010).

**Fig. 1:**
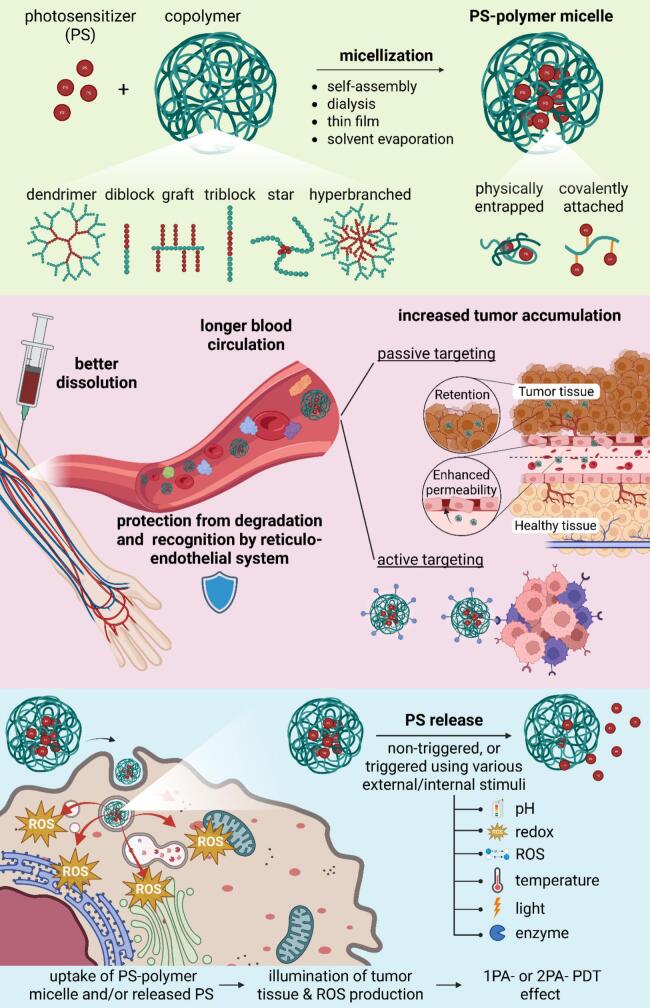
A schematic illustration of this review's scope is shown, covering the synthesis of polymer materials used as micellar carriers, the different approaches to photosensitizer encapsulation/conjugation into these systems, which leads to better dissolution, longer blood circulation, protection of PS and increased tumor accumulation, and finally the various internal/external stimuli, that can be used for spatio-temporal controlled release of photosensitizers in the affected tumor area.

Unlike other reports on PDT, the present work is conceptualized as comprehensive perspective, examining the entire continuum of polymeric micelle-based PDT – from polymer synthesis and micelle design to photosensitizer incorporation, controlled release and therapeutic applications. Great focus is given on unique potential of micellar carriers to overcome limitations of traditional PSs and to advance the therapeutic efficacy of PDT. The overall scope of this review is illustrated in Fig. 1.

## 2 Carriers based on polymer micelles

### 2.1 Building blocks

Polymer micelle carriers are commonly used vehicles for the delivery of a wide range of drugs, offering the potential to improve their therapeutic effect. Micelles are formed by spontaneous supramolecular self-assembly of typically di- (hydrophilic-hydrophobic) or tri- (hydrophilic-hydrophobic-hydrophilic) block or graft copolymers under aqueous conditions, above a concentration known as the critical micelle concentration (CMC). (Matsumura and Maeda, 1986) The micelle formation is driven by the poor compatibility of the hydrophobic segment with the aqueous phase, and its preferential assembly into the core, while being surrounded by a hydrophilic block, stabilizing the interface and creating a corona around the hydrophobic core, resulting in a typical core-shell like structure.

The hydrophilic shell's main role is to stabilize and solubilize the micelle. Several hydrophilic polymeric materials are commonly used, with poly(ethylene glycol) (PEG) being the golden standard. Benefiting from low costs, its biocompatibility, and colloidal stability during blood circulation, it is recognized as a safe delivery system for medical applications by the US Food and Drug Administration (FDA) and, as such, has been clinically used for breast and ovarian cancer treatment (Barenholz, 2012). In recent years, however, the phenomenon of anti-PEG antibodies has emerged (Kozma et al., 2020), creating a major obstacle to effective drug delivery using PEG-based systems. In addition, it has been shown that PEG-liposomes are rapidly cleared at low lipid doses and upon repeated administration as well, leading to the accelerated blood clearance (ABC) phenomenon (Ishida and Kiwada, 2008). To prevent the recognition by the host immune system, “dePEGylation” techniques have been proposed to trigger the shedding of PEG from the carrier. (Kong et al., 2019) To further solve the problem, various alternative hydrophilic polymers to PEG, such as poly(*N*-vinyl-2-pyrrolidone) (Gjuroski et al., 2019; Le Garrec et al., 2002; Rybkin et al., 2023), poly(2-oxazoline)s (Huntošová et al., 2021; Oudin et al., 2019; Shieh et al., 2010), polyacrylamides and their derivatives, or natural polysaccharides (Breitenbach et al., 2019a; Debele et al., 2017; Naveen and Shastri, 2019; Wang et al., 2018b) have been synthesized.

Poly(N-vinyl-2-pyrrolidone) (PVP) is a widespread amphiphilic polymer with applications mainly in drug formulations due to its good water solubility, high biocompatibility and FDA approval. A formulation system of PVP with trisodium salt of chlorine e6, (Ce6-PVP), also known as Photolon™, has been long studied for head and neck cancer and skin melanomas. (Copley et al., 2008) In addition, its topical application can improve endoscopic detection of cancerous lesions (Ganzleben et al., 2020) W.W.L. Chin et al. demonstrated the ability of the Photolon™ formulation to enhance to PDT efficacy in the human bladder cancer (Chin et al., 2008; Chin et al., 2007).

Poly(2-oxazoline)s, especially poly(2-methyl-2-oxazoline) (PMeOx) and poly(2-ethyl-2-oxazoline) (PEtOx), constitute another potential PEG replacement, as they also exhibit stealth and non-fouling properties. Altering the length of the side chain, their water-solubility can be altered, offering the possibility to fine-tune final polymer's physiological behavior as needed. (Luxenhofer et al., 2010).

Poly(*N*-(2-hydroxypropyl)methacrylamide) (HPMA) is another water-soluble, biocompatible, and non-fouling material able to circulate without eliciting the ABC effect upon repeated administration. Importantly, the HPMA copolymers allow for the conjugation of anticancer drugs and/or targeting moieties to the same polymer chain. (Kierstead et al., 2015).

A novel and interesting group of hydrophilic polymers, with potential application as a micellar shell layer is represented by zwitterionic polymers with an equal number of cations and anions along their chain. Their non-fouling properties result from interactions with water via strong ion-dipole electrostatic interactions, creating hydration layers able to repel biological molecules. As such, they differ from PEG, whose hydration layer is created by hydrogen bonding with water. (He et al., 2008; Izquierdo-Barba et al., 2016; Wu et al., 2012) Despite the intriguing properties of zwitterionic polymers, there are only a few mentions of studies involving their interactions with photosensitizers for potential application in PDT and tumor imaging. (Chen et al., 2020; Chen et al., 2016; Gjuroski et al., 2021; Vermathen et al., 2013; Wang et al., 2020).

Synthetic polymers typically used as the hydrophobic segment of a micellar structure include polystyrene, polybutylene oxide (PBO), polybutadiene (PB), polymethylacrylate (PMA) or poly(propylene oxide) (PPO). (Kuperkar et al., 2022; Pitto-Barry and Barry, 2014) Other hydrophobic materials, especially poly(Ɛ-caprolactone) (PCL) or poly(α-hydroxy esters), are extensively used thanks to their biocompatibility, affordability, and especially, biodegradability. (Bartnikowski et al., 2019; Mir et al., 2017) Both are FDA-approved for specific biomedical applications, poly(α-hydroxy esters) are also approved by the European Medicines Agency (EMA) (Chakraborty et al., 2024; Mir et al., 2017). Incorporating biodegradable or environmentally stimuli-responsive material into the micellar structure can further enhance the efficacy of the treatment. Poly(*N*-isopropylacrylamide) (PNIPAM) is a well-known polymer for its thermo-responsivity, exhibiting phase transition properties. Moreover, it is a biocompatible and non-toxic material, making it an ideal candidate for drug delivery systems. PNIPAM-based hydrogels are extensively used for various purposes, such as wound dressing. (Throat and Bhattacharya, 2024a) Chemically cross-linked PNIPAM/photosensitizer hydrogels are employed in photodynamic therapy as well. (Belali et al., 2018) Responsive materials and the mechanism of photosensitizer release will be discussed in more detail later on.

Besides synthetic polymer materials, natural biopolymers based on polysaccharides or poly(amino acids) represent an intriguing group of building blocks for micelle delivery systems. Polysaccharides can be neutral or charged with varying structures and molecular weights. (Hsu et al., 2024) Even though poor processability often hinders their application in native form, derivatization of various functional groups can be employed. (Atanase, 2021) In the realm of photodynamic therapy, hydroxypropyl cellulose (HPC)-derived hydrogels have been used for topical application of photosensitizers, ideal for actinic keratosis or non-melanoma skin cancers. (Burloiu et al., 2024) Polysaccharides can also be used as tumor targeted moieties or stimuli responsive carriers. (Huang et al., 2014; XWang et al., 2018b).

Similarly, amino acids offer versatility and flexibility in their structure, enabling to the fabrication of specific types of peptide chains with controllable characteristics. Indeed, self-assembled peptides are receiving a lot of attention in drug delivery due to their biocompatibility, biosafety and bioavailability. (Sheehan et al., 2021) Moreover, thanks to their natural character, some already known sequences can enter the cell without damaging the integrity of cellular membranes, constituting a class of cell penetrating peptides (CPPs), or can be used as targeting peptides or stimuli responsive linkers. (Berillo et al., 2021).

### 2.2 Micellar structure

Block copolymers are an interesting class of polymeric materials thanks to their ability to form a variety of structures, such as linear diblock and triblock copolymers, star copolymers, hyperbranched, dendrimeric copolymers or graft copolymers. Amphiphilic copolymers comprising hydrophilic and hydrophobic segments, are able to self-assemble in solvents selective for one of the blocks. The insoluble block forms the inner core, whereas the soluble block forms the corona around it. The phenomenon of micellization has been studied for decades, from both theoretical and experimental points of view. (Hadjichristidis et al., 2024).

Parameters such as the hydrophobic/hydrophilic block ratio, chemical characteristics, or the molecular weights of each block influence the CMC and micelle stability. Even though micelles are often depicted as spherical, solid particles, depending on aforementioned variables, they can vary in terms of size and shape. For, a more in-depth evaluation of micelle stability, see the review by Shawn C. Owen. (Owen et al., 2012).

Diblock copolymer micelles, consisting of two covalently bonded polymer blocks (AB), are the most commonly used and extensively studied carriers in PDT. This popularity stems from their straightforward synthesis, tunable self-assembly, as well as their well-understood behavior in aqueous environments. Their molecular simplicity makes them highly versatile: by varying the length and composition of each block, researchers can finely control micelle size, drug loading capacity, and release kinetics. This tunability also allows for the introduction of functional groups to impart responsiveness to stimuli or targeting capability. Their adaptable properties continue to make diblock copolymers a mainstay in the field of drug delivery. (Hussein and Youssry, 2018).

Triblock copolymers, comprising three polymer segments (ABA or ABC arrangements) can offer additional versatility, however, their synthesis is more complex and their physicochemical behavior can be harder to predict or control, which may limit their widespread adoption compared to diblock copolymers. Nevertheless, triblock copolymers comprising of polyoxypropylene (PPO) and polyoxyethylene (PEO) as the hydrophobic and hydrophilic units, respectively, are commercially available and FDA-approved under the name Pluronics®. Their versatility lies in the possibility of obtaining carriers with different shapes and sizes, e.g. micelles, rods, lamellar, hexagonal, dendrimers, etc., by simply changing the reaction temperature, concentration of monomers used, and the composition of the blocks, i.e. the chain length. (Kumar et al., 2023) As a result, these triblock copolymers are more frequently employed as drug carriers than their PEO-PPO diblock counterpart. (Herzberger et al., 2016).

In comparison with block copolymer-based micelles, micelles constructed from graft copolymers often exhibit a more complicated and entangled polymeric structure. The self-assembly of graft copolymers can be controlled by careful selection of the polymerization degree of graft chains, graft density, as well as bulkiness of the monomer units. With the development of precise polymer synthesis techniques, the synthesis of graft copolymers, using either ´grafting-(on)to´, ´grafting-through´ or ´grafting-from´ approaches, has become easier. (Yokoyama et al., 2018) Nevertheless, compared to block copolymers, the research on self-assembling graft copolymer has been limited, and the prediction of the final assembled structures is somewhat complicated. (Sakamoto and Nishimura, 2022).

Sometimes, simply attaching a hydrophobic photosensitizer to a hydrophilic polymer carrier is enough to favor micellar formation in aqueous conditions. Tavares et al. fabricated polymeric micelles using water soluble biocompatible HPMA-based copolymers as carriers of covalently attached pyropheophorbide-a 5-hydroxy-2-pentanone ester (dPyF). The hydrophobic core is then made up of the stacked photosensitizer, wrapped by the hydrophilic carrier. (Tavares et al., 2023) Similarly, Gomes et al. used the Heck reaction to incorporate novel β-substituted triazole-porphyrin derivatives (TZ-PORs) into polyvinylpyrrolidone (PVP) micelles. (Gomes et al., 2020).

Star polymers, as complex structures with at least three chains (arms) radiating from one central core, have been extensively studied as drug carriers, especially for their unique topological structures and properties. Using different synthetic approaches, regular star polymers, with the same arm segments made up of homopolymers or copolymers, as well as irregular and asymmetric “miktoarm” architectures with varying structures, molecular weights, or functional groups, can be designed. (Wu et al., 2015).

From the point of view of delivery into tumorous tissue, it is necessary for the carrier to exhibit sufficiently high stability (hence low CMC) and to prevent the premature release of loaded or bound PS during transportation. Not all combinations of materials and PS are suitable for effective cancer treatment. Some systems may exhibit low loading capacity, poor stability of the resulting micellar structures, or, conversely, rapid release (burst release), leading to undesirable effects outside the targeted tissue. The final behavior of the system thus reflects the interplay between the photosensitizer and the polymeric carrier, specific to each individual case. (Gibot et al., 2014; He et al., 2023).

### 2.3 Photosensitizer incorporating strategies

The encapsulation of PSs within the micellar core is a pivotal strategy in enhancing the delivery of PSs and the PDT efficacy within the tumor site. The choice of micellar structure significantly influences the loading efficiency, stability, and release kinetics of the PSs. There are several ways of incorporating hydrophobic drugs inside the micellar core, such as (i) simple physical encapsulation or (ii) covalent binding to the polymer molecules. (Yakavets et al., 2019) Each has distinct advantages and are suited to different types of PSs and polymer structures.

#### 2.3.1 Encapsulation

Physical entrapment or loading, utilizing mainly hydrophobic or electrostatic interactions between the drug and the hydrophobic core, is a very straightforward approach for encapsulation of hydrophobic drugs into the micelle structure. The most commonly used methods involve oil-in-water (Miller et al., 2013; Sant et al., 2005) or water-in-oil-in-water double emulsion (Avgoustakis et al., 2002) techniques, direct dialysis (Van Butsele et al., 2009), co-solvent evaporation (Aliabadi et al., 2007; Šmejkalová et al., 2017) or thin-film rehydration (Ai et al., 2014), and freeze-drying/lyophilization (Esparza and Onyuksel, 2019). The chosen method can significantly affect the physicochemical parameters of the final micellar system as well as the drug encapsulation efficiency. In the realm of PDT, thin film hydration, solvent evaporation and dialysis are the most commonly used methods for encapsulating hydrophobic PSs within micellar systems.

The thin film hydration method enables the partitioning of PS into the core by forming a thin polymer-drug film, which facilitates uniform micelle self-assembly with high loading efficiency upon hydration. The thin film method was employed by Lamch et al. to fabricate polymeric micelles based on amphiphilic copolymer, composed of hydrophilic PEG and hydrophobic PCL or poly(D,l-lactide) (PDLLA), loaded with *tert*-butyl zinc(II) phthalocyanine (ZnPc-*t*-but_4_). Their approach yielded micelles with average hydrodynamic diameters (*D*_H_) of ∼40 nm (mPEG-*b*-PCL) and ∼ 25 nm (mPEG-*b*-PDLLA) with relatively low polydispersity indexes (PDI) (PDI between 0.09 and 0.27 for both systems). The loading efficiency for both studied systems was approximately 100 %, reaching PS/polymer ratios of 15.3 % (ZnPc-*t*-but_4_ loaded mPEG-*b-*PCL) and 10.0 % (ZnPc-*t*-but_4_ loaded mPEG-*b*-PDLLA), respectively. (Lamch et al., 2021) Using thin film procedure, Wennink et al. were able to encapsulate m-tetra(hydroxyphenyl)chlorin (mTHPC) inside micelles based on benzyl-poly(Ɛ-caprolactone)-*b*-methoxy poly(ethylene glycol) (Ben-PCL_7_-mPEG_45_) with a maximal loading capacity of 17 wt%. In spite of such high concentrations of hydrophobic photosensitizer, no signs of aggregation inside the micelles were observed. Such aspect is very important, since aggregation of photosensitizers often hampers ^1^O_2_ generation. (Wennink et al., 2017).

Although thin film hydration and solvent evaporation are often (mis)interpreted as the same, these two approaches slightly differ. The solvent evaporation method involves the dissolution of both copolymer and the photosensitizer in organic solvent, which is then added, usually dropwise, into the aqueous phase. The organic solvent is then evaporated. In contrast to the thin film hydration method, where micelles are formed upon water interaction with the dry polymer-PS film, in solvent evaporation approach, the micelles are formed as the organic solvent evaporates, forcing the polymer and hydrophobic photosensitizer to assemble. (Negut and Bita, 2023) Ding et al. encapsulated 5,10,15,20-tetrakis(*meso*-hydroxyphenyl) porphyrin (mTHPP) into PEG-*b*-poly(D,l-lactide) (PEG-*b*-PLA) and poly(ethylene glycol)-*b*-poly(2-(diisopropylamino)ethyl methacrylate (PEG-*b*-PDPA) micelles by co-dissolving them in tetrahydrofuran and then adding the mixture dropwise into MilliQwater under sonification. THF was allowed to evaporate overnight. The resulting m-THPP-loaded micelles were further purified by centrifugation dialysis to remove unreacted components. (Ding et al., 2011b).

Dialysis is considered a gentler, though more time-consuming, approach to micelle formation. The copolymer and photosensitizer are dissolved in a common solvent, usually *N*,*N*-dimethylformamide (DMF), dimethyl sulfoxide, acetone, etc., followed by the addition of aqueous solvent to stimulate the micelle formation. The mixture is then dialyzed against water to remove the organic solvent. (Kotta et al., 2022) Obata et al. used the dialysis approach to evaluate the effect of hydrophobic segment of eight amphiphilic block copolymers, based on *N*-substituted polyacrylamide and poly(polyethylene glycol monomethyl ether acrylate) (PPEGA) on formation of ZnPc bearing micelles. Individual copolymers were dissolved with ZnPc in DMF and phosphate-buffered saline (PBS) was added dropwise, causing aggregation of the hydrophobic segments and the photosensitizer into ZnPc-loaded micelles. The mixture was then dialyzed thoroughly with PBS. (Obata et al., 2021).

The undeniable benefits of physical entrapment of hydrophobic drugs are its simplicity and applicability to a wide range of hydrophobic molecules, including those that do not have suitable functional groups for covalent bonding. However, the necessity of using organic solvents, and the subsequent need for its removal partially reduces those benefits. For polymers with good water solubility, the direct dissolution approach is the most appropriate, as the polymer self-assembles into micelles with the aid of continuous stirring. (Kotta et al., 2022) This strategy is commonly employed for the micellization of polyion complexes (PIC). The group of Kataoka dedicated their research to the study of PIC micelles formed by electrostatic interactions between negatively or positively charged dendrimeric Zn-porphyrins and oppositely charged block copolymers. (Stapert et al., 2000) (Zhang et al., 2003) The dendrimeric structure of the porphyrin prevents the formation of aggregates due to the large bulky dendritic wedges, resulting in an order-of-magnitude enhancement in photodynamic efficacy. (Jang et al., 2006).

Physical encapsulation of PSs within polymeric micelles offers a promising strategy for targeted delivery in PDT, but it is accompanied by several critical limitations that affect therapeutic efficacy and clinical translation. One of the most prevalent issues is low drug loading capacity, since the confined core of the micelles often struggles to accommodate sufficient quantities of PS. To further increase the loading capacity, it is possible to modify the micellar core, e.g. by incorporating more hydrophobic segments for enhanced interactions with loaded PS. Polymeric micelles based on Ben-PCL_7_-mPEG_45_ showed a maximal loading capacity of 17 % for mTHPC at a feed ratio of 20 %. Higher feed ratio of 30 % did not result in further increase in loading. Employing Ben-PCl_5_-PEG_17_ with higher content of hydrophobic PCL (43 wt% instead of 28 wt%) further enhanced the loading capacity to 30 wt% at 30 % feed ratio of mTHPC. (Wennink et al., 2017).

**Fig. 2:**
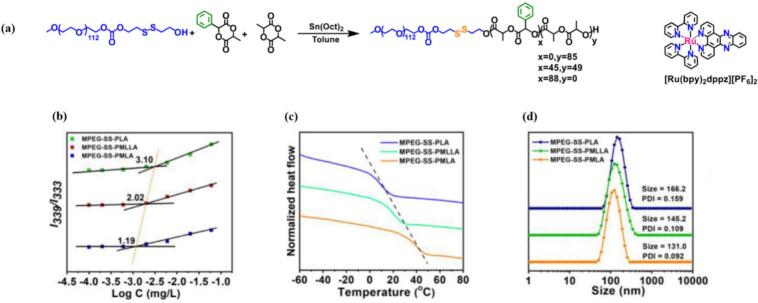
(a) the synthetic process of the amphiphilic phenyl-functionalized poly(ethylene glycol) polylactide block copolymers and the hydrophobic ruthenium(II) polypyridyl complexes; (b) CMC determination plot of the intensity ratio of *I*_339_/*I*_333_ from pyrene excitation spectra versus the logarithm of copolymers´ concentration, (c) DSC spectra, (d) the diameter measured by DLS for MPEG-SS-PLA, MPEG-SS-PMLLA and MPEG-SS-PMLA. Reproduced with permission from (He et al., 2023). Copyrights © 2023 Elsevier B.V.

Furthermore, increasing the interaction between the micellar core and encapsulated PS can enhance not only the thermodynamic driving force for the drug loading, but also the micellar stability. He et al. synthesized a set of self-assembling micellar carriers based on phenyl-functionalized poly(ethylene glycol) polylactide block copolymers (MPEG-SS-PMLA) for the ultra-high loading and redox-responsive release of Ru(II) polypyridyl complexes (see Fig. 2 (a)). The hydrophobic core was precisely built by polymerizing lactide (LA) and phenyl-functionalized cyclic lactide (MLA) monomers at different ratios, in order to increase the π- π stacking interaction with the Ru complex. The three copolymers MPEG-SS-PLA, MPEG-SS-PMLLA and MPEG-SS-PMLA with LA/MLA feed ratios of 100/0, 50/50 and 0/100 respectively, were amphiphiles, able to self-assemble into micelles in aqueous conditions. Their CMC gradually decreased with the increasing number of phenyl moieties on the PLA side chains, reaching a CMC of 1.19 mg L^-1^ for MPEG-SS-PMLA (Fig. 2 (b)). The positive influence of phenyl groups on the overall stability of the system was further supported by differential scanning calorimetry (DSC) and dynamic light scattering (DLS) measurements, in which MPEG-SS-PMLA exhibited the highest glass transition temperature (*T*_g_) (Fig. 2(c)) and the smallest mean hydrodynamic diameters (Fig. 2(d)), suggesting the strongest hydrophobic intermolecular interactions. As a result, both the loading content (14.4 %) and encapsulation efficiency (84.3 %) were improved compared to non-functionalized MPEG-SS-PLA carrier (loading content 9.7 % and encapsulation efficiency 53.7 %). (He et al., 2023).

Additionally, the micellar systems with loaded PS often face premature drug release, usually stemming from weak, non-covalent interactions between the hydrophobic core of the micelle and the PS. This phenomenon can result in unintended release during systemic circulation, diminishing selective accumulation in tumors and increasing the risk of off-target phototoxicity. Taken together, while physical encapsulation provides a relatively simple and versatile platform for PS delivery, more advanced delivery strategies – such as covalent conjugation, stimuli-responsive materials, or hybrid micelle systems - may further enhance the stability, bioavailability, and therapeutic efficacy of PSs in PDT. (Dantas et al., 2022).

#### 2.3.2 Covalent binding

Covalent binding of PS to polymer micelles offers several key advantages that enhance the system's stability and the efficacy of PDT. By forming a stable covalent linkage, the PS remains securely attached to the carrier, minimizing burst release and reducing the likelihood of off-target effects, improving the pharmacokinetic profile, prolonging circulation and improving accumulation within tumorous tissue. (Dantas et al., 2022).

**Fig. 3:**
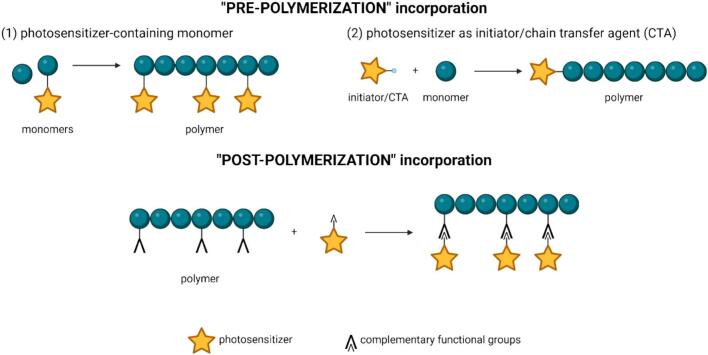
Techniques of photosensitizer incorporation into the polymer system.

There are several ways (see Fig. 3) to proceed when designing a PS-containing micellar carrier using covalent linkage. First, “pre-polymerization” incorporation entails less conventional approaches, such as copolymerizing PS-containing monomer, or using an initiator or chain transfer agent (CTA), linked with PS molecules. Conversely, the more widely exploited approach involves “post-polymerization” conjugation of PS to pre-formed micelles, taking advantage of complementary functional groups on the polymer chain and the PS molecules.

##### 2.3.2.1 “Pre-polymerization”

The copolymerization of PS-containing monomers has emerged as a powerful strategy for engineering polymeric micelles tailored for applications such as PDT and bioimaging. By tethering a polymerizable group (e.g., methacrylate (Ballestri et al., 2018), vinyl (Pechnikova et al., 2014), norbornene (Ahmetali et al., 2023; Kimura et al., 2002) or styrene (Makhseed et al., 1999)) to a PS molecule, one can *co*-polymerize it with hydrophilic and hydrophobic monomers to form defined block copolymers. Several polymerization methods have been explored, including conventional free radical polymerization (FRP) (Makhseed et al., 1999; Pechnikova et al., 2014), controlled radical techniques like reversible addition−fragmentation chain-transfer polymerization (RAFT) (Xu et al., 2015a), or ring-opening methods such as ring-opening metathesis polymerization (ROMP) (Kimura et al., 2002). While monosubstituted derivatives of PSs are the most suitable for obtaining porphyrin-containing linear polymers, multifunctional PSs can produce crosslinked networks with applications in biomedicine, electrochemistry, and sensors. (Çetinkaya et al., 2021; Pechnikova et al., 2014).

While the polymerization of PS-containing monomers represents an elegant approach to the preparation of micellar structures, achieving a precisely defined copolymer can be challenging without a thorough understanding of the monomer's behavior, requiring complex and detailed characterization. It is well known, that reactivity and copolymerization efficacy of the monomer are strongly influenced by its structure. Factors such as the stabilization of the propagating center, or the presence of electron-donating or electron-withdrawing substituents can play a critical role. Additionally, steric hindrance caused by bulky substituents can significantly affect the polymerization rate, and consequently, the overall outcome of the process. (Prokopová, 2007) The addition of long spacer between the photosensitizer and the monomer unit can help to minimize the detrimental steric effect of the polymerization reaction due to the large macrocycle (Makhseed et al., 1999).

A lot of fascinating research has been performed in the area of photosensitizers-based initiators. Taking advantage of a series of functional groups (such as -OH, -COOH, -NH_2_), they can be derivatized and used in ring opening polymerizations (ROP) or controlled/living radical polymerizations yielding polymers with diverse topologies and structures. The porphyrin-initiators, such as (5,10,15,20-tetraphenylporphinato)aluminum chloride or its alkoxide analogue, have been known and employed since the 1980s for ROP of epoxides, β-lactone or Ɛ-caprolactone, yielding polymers with controlled molecular weight and narrow distribution. (Endo et al., 1987) Zhang et al. used a protoporphyrin IX (PpIX) derivative bearing two 2,2´-(ethylenedioxy)bis(ethylamine) (TEG) chains for the preparation of PpIX-PCL-PEG polymer micelles for subsequent loading of doxorubicin (Dox). The initiator not only led to well-defined polymers with narrow dispersity, but it further increased the stability of the formed micelles due to its π- π interactions. (Zhang et al., 2016) In case of atom transfer radical polymerization (ATRP), the copper complexes typically used as catalysts, can easily coordinate with free base porphyrin initiators. Therefore, inserting a metal as central atom can help to avoid the partial metalation of the core during polymerization (de Loos et al., 2005; High et al., 2007). The metalation of photosensitizers can in addition increase the photodynamic therapy efficacy. Due to the “heavy atom effect” the rate of intersystem crossing towards triplet state formations can be favored, introducing the possibility of increasing the ^1^O_2_ generation (Arnaut, 2011; Gorman et al., 2004).

**Fig. 4:**
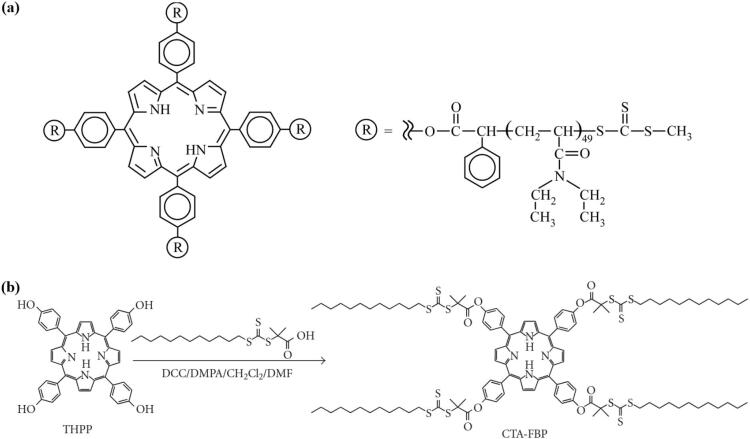
(a) Tetra-functional porphyrin containing trithiocarbonate groups were synthesized by simple esterification method and employed as CTA to prepare well-defined 4-arm star-shaped polymers based on *N,N*-diethylacrylamide (PDEA). Reproduced with permission from (Yusa et al., 2009). Copyright © 2009 Wiley Periodicals, Inc. (b) Synthesis of porphyrin cored RAFT agent (CTA-FBP). Reproduced from (Wu et al., 2011). Copyrights © 2011 Lin Wu et al. CC BY 3.0.

Additionally, PSs and their derivatives can be modified into CTAs for RAFT polymerizations. Tetra-functional porphyrin containing trithiocarbonate groups were synthesized by a simple esterification method and employed as CTA to prepare well-defined 4-arm *star*-shaped polymers based on *N,N*-diethylacrylamide (PDEA) (Fig. 4 (a)). (Yusa et al., 2009) Free-base 5,10,15,20-tetrakis(4-hydroxyphenyl)-21H,23H-porphine (FBP) was transformed into CTA-FBP by attaching S-1-dodecyl-S´-α, α´-dimethyl- α´´-acetic acid (Fig. 4 (b)) and later used for preparation of porphyrin cored homopolymers and amphiphilic block copolymers with star-like and flower-like micellar structures. (Wu et al., 2011).

In general, the majority of PS-based initiators or CTAs are used to create branched or star shaped polymers. This stems from the fact, that monofunctionalized PS requires more complex preparation and purification operations as opposed to their tetra-functional analogues. Even though monofunctional PSs are commercially available, their price reflects the complexity of their synthesis. (High et al., 2007).

##### 2.3.2.2 “post-polymerization”

Post-polymerization covalent attachment of photosensitizers is a versatile and often preferable approach in the design of polymeric materials for PDT. It refers to the chemical modification of polymers after their synthesis by attaching the PS molecules onto the polymer backbone or side chains. This approach provides flexibility and modularity in the design of functional materials, and allows the incorporation of PS without interfering with the polymerization process itself. In addition, due to the usually mild reaction conditions, there is a lower risk of PS degradation, since the PS is notexposed to increased temperatures or radicals as is during polymerization.

**Fig. 5:**
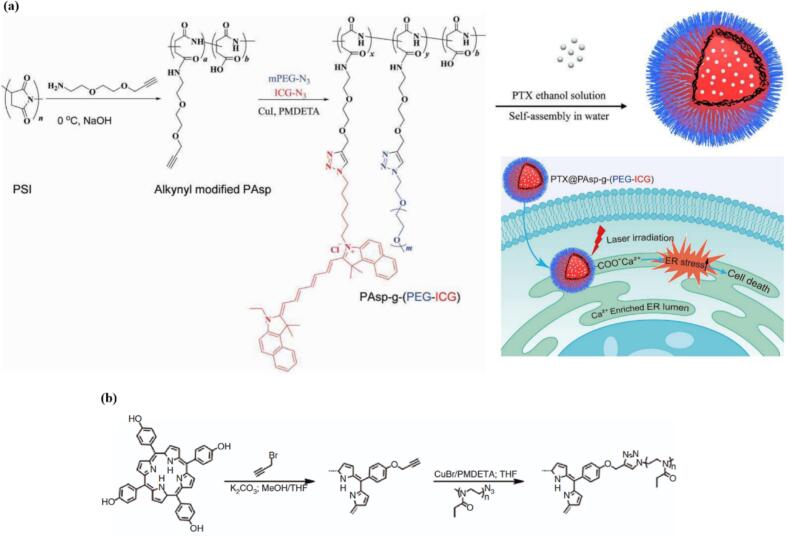
(a) detailed preparation procedure of the comblike PAsp-*g*-(PEG-ICG) micelles loaded with PTX (PTX@PAsp-*g*-(PEG-ICG) accompanied by illustration of ER targeting process and mechanism of cell death. Micelles accumulate in the ER lumen through coordination affinity of the Ca(II) ions to the carboxyl groups PAsp, Upon laser irradiation of ICG, the generated ROS would lead to elevated ER stress and induce cancer cell death. Reproduced with permission from (Wan et al., 2018). Copyrights © 2018 The Royal Society of Chemistry. (b) Alkyne modification of [TPP-OH]_4_ via esterification with propargyl bromide and subsequent CuAAC chemistry with PEtOx_5_-N_3_, forming porphyrin-centered star-shaped [TPP-PEtOx_5_]_4_. Reproduced from (Rudolph et al., 2015). Copyrights © 2015 by De Gruyter. CC BY-NC-ND 3.0.

Among the possibilities are click reactions, particularly copper-catalyzed azide–alkyne cycloaddition (CuAAC) and strain-promoted azide-alkyne cycloaddition (SPAAC). These reactions require mild, simple reaction conditions and offer high efficiency and chemical yields. Additionally, the copper-free variant SPAAC overcomes the potential cytotoxicity associated with copper contamination. (Chan et al., 2013) Wan et al. rationally designed and successfully prepared PAsp-*g-*(PEG-ICG) micelles by grafting azido-modified polyethylene glycol and azido-modified photosensitizer indocyanine green (ICG) onto alkynyl-modified poly(aspartic acid) (PAsp) through the CuAAC. They were able to further encapsulate paclitaxel with high loading content, with up to 28 % drug loading. Their systems, designed for synergistic chemo- and photodynamic therapy, were able to target endoplasmic reticulum, offering a promising strategy for enhanced cancer cell ablation see (Fig. 5(a)). (Wan et al., 2018) Rudolph et al. used CuAAC for the synthesis of star-shaped PEtOx featuring a porphyrin core. In the first step of their multi-step synthesis, they used the hydroxyl groups on commercially available 5,10,15,20-tetrakis(4-hydroxyphenyl)-21H,23H-porphyrin for the alkyne-introduction by etherification with propargyl bromide. For the synthesis of azide-functionalized PEtOx, (PEtOx_5_-N_3_) the monomer was polymerized via microwave-assisted cationic ring opening polymerization (CROP) using methyl *p*-toluenesulfonate (MeTos) and terminated by the addition of sodium azide, leading to a substitution reaction and introduction of azide end groups. Using CuAAC, with an excess of PEtOx_5_-N_3_ compared to alkyne-functionalized porphyrin to ensure full conversion, the final symmetrical star-shaped porphyrin-centered [TPP-PEtOx_5_]_4_ was synthesized (see Fig. 5(b)). (Rudolph et al., 2015) Cycloaddition can also be used for a “click” crosslinking between encapsulated alkynyl-containing porphyrins and azido-functionalized block copolymers. (Xu et al., 2014).

Despite multiple reports of cycloaddition employment (Rudolph et al., 2015; Wan et al., 2018; Xue et al., 2019; Xu et al., 2014), more frequently used approach involves carbodiimide chemistry. Although primarily utilized in peptide synthesis to facilitate the formation of amide bonds between amino (–NH₂) and carboxyl (–COOH) groups of amino acid residues, this approach can also be effectively applied for the conjugation of photosensitizers to peptide-containing delivery systems. Choi et al. conjugated chlorine e6 (Ce6) to poly-l-lysine grafted with monomethoxy-poly(ethylene glycole) using 1-ethyl-3-(3-dimethylaminopropyl)carbodiimide hydrochloride (EDC), resulting in Ce6-containing macromolecules sensitive to tumor-associated proteases. (Choi et al., 2006a, Choi et al., 2006b) A popular approach for the conjugation of PS to polymer backbone involves the use of additives, such as *N*-hydroxysuccinimide (Campo et al., 2007; Huang et al., 2016) or pentafluorophenol (Fang et al., 2018; Young et al., 2024), *N*-hydroxybenzotriazole (Kiew et al., 2017; Li et al., 2017a), to prevent or suppress the formation of *N*-acylurea. Corresponding active esters of additives may be less reactive intermediates, but they can increase the efficiency of carbodiimide-mediated reactions, easing the formation of covalent bonds. Moreover, these active esters can often be prepared in advance, purified and stored. (El-Faham and Albericio, 2011).

In addition to facilitating stable amide bond formation, carbodiimide chemistry can be also employed for the formation of labile ester bonds, through the reaction of an activated carboxylic acid with a hydroxyl group. This esterification process typically involves the activation of the carboxyl group using carbodiimide reagents such as EDC or *N*,*N´*-Dicyclohexylcarbodiimide (DCC), forming a reactive *O*-acylisourea intermediate. The intermediate can then undergo nucleophilic attack by a hydroxyl group, resulting in the formation of a covalent ester linkage. Even though alcohols are in general much poorer nucleophiles than amines, and the degree of undesired *N*-acylurea formation is subsequently greater than in amide formations, the addiction of 4-Dimethylaminopyridine (DMAP) in catalytic amounts can compensate for this tendency. (Tsakos et al., 2015) Kumari et al. conjugated Ce6 to mPEG-PLA via the distal hydroxyl groups of PLA chain and free carboxyl groups in Ce6 through an ester bond. This ester bond between the drug and polymer backbone is labile to the abundant esterase activity in the cells. (Kumari et al., 2019).

**Fig. 6:**
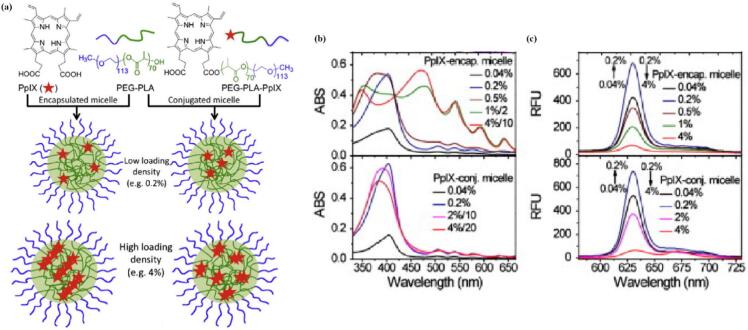
(a) schematic illustration of two different types of PpIX/PEG-PLA micelles from non-covalent encapsulation and covalent conjugation methods. At low loading density (i.e. 0.2 %) PpIX exists as monomers in both micelle formulations; at high loading density (i.e. 4 %), PpIX is in form of aggregates or dimers in encapsulated and conjugated micelles respectively.; UV/VIS (a) and fluorescence (λ_ex_ = 404 nm) (b) of PpIX-encapsulated and PpIX-conjugated micelles. Reproduced with permission from (Ding et al., 2011a). Copyrights © 2011 Elsevier B.V.

Indeed, a lot of effort in PS- micellar carriers have moved towards the covalent attachment via stimuli responsive linkers, or encapsulating PS into stimuli responsive carriers in order to ensure stable drug retention during systemic circulation, only to be subjected to specific environmental stimuli at the target site. This approach enables space and time-controlled release kinetics, resulting in more specific and sustained drug release upon accumulation. The individual release mechanisms, as well as the various stimuli, that can be utilized, will be discussed later on. It is important to remember, that the selection of entrapment mode (covalent conjugation vs physical encapsulation) can influence the way PS molecules are arranged within the hydrophobic core, subsequently affecting the fluorescence efficiency, ^1^O_2_ quantum yield and overall PDT efficacy. Ding et al. prepared two series of PpIX-containing PEG-*b*-PLA spherical micelles using either non-covalent hydrophobic encapsulation strategy or covalent conjugation to the hydroxyl group of PEG-*b*-PLA through ester linkages (Fig. 6 (a)). In both series, the PpIX loading density (weight percentage of PpIX over micelles) varied from 0.04 % to 4 %. They systematically examined the UV–vis absorption, fluorescence emission, ^1^O_2_ yield and PDT efficacy. At low PpIX loading density (less than 0.2 %), PpIX remained in the monomeric state, indicated by a sharp Soret band at 404 nm, strong fluorescence intensity and high ^1^O_2_ quantum yield, regardless of incorporation strategies. With increasing the loading density (i.e. 4 %), the entrapment mode had dramatic effects on the PS´ state in the micelles. In case of encapsulated micelles, PpIX formed aggregates, indicated by the split Soret bands and complete quenching of fluorescence and relative ^1^O_2_ quantum yield (ɸ_Δ_) (ɸ_Δ_ = 0.06).^1^ In comparison, the conjugated micelles, the PpIX was present as dimers, retaining considerable ^1^O_2_ generation capacity (ɸ_Δ_ = 0.48)^1^ (Fig. 6 (b)). (Ding et al., 2011a).

In general, physical encapsulation of PSs into micelles is simple and straingforward approach, relying on physical interactions within the hydrophobic core. Nonetheless, it often suffers from premature release and aggregation at higher loading, which in turn reduces singlet oxygen generation. By contrast, covalent conjugation (either pre- or post-polymerization) typically requires more complex chemistry, but ensures superior stability, controlled release, and low/none premature loss of PS during transport. This improved stability translates into prolonged blood circulation, reduced off-target losses during transport, and consequently enhanced accumulation at the tumor site, resulting in a more reliable and efficient PDT outcome.

### 2.4 The benefits of micellar carriers for delivery of PS

**Fig. 7:**
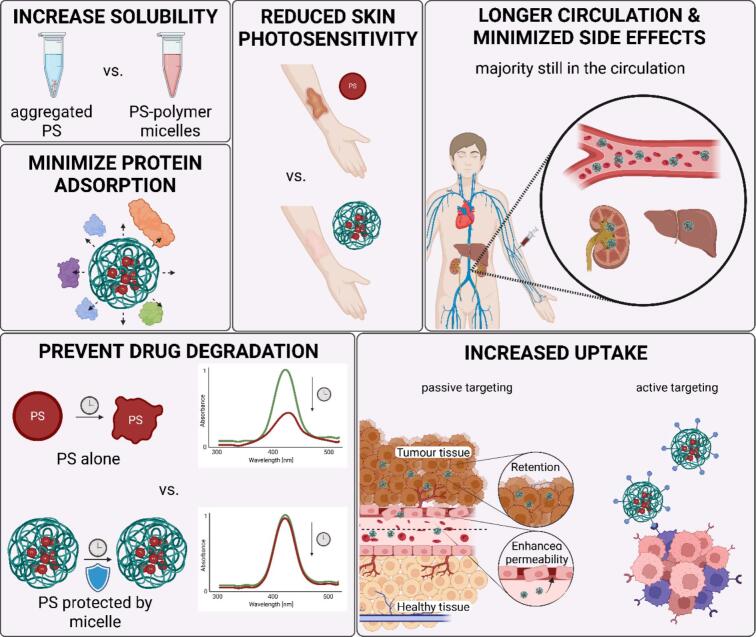
The benefits of the use of micelle for photosensitizer's delivery.

Upon intravenous administration of free PS, as single molecule or as a solvent- or excipient-based formulations, redistribution and interactions with blood serum proteins, such as albumin, or low- (LDL) and high-density lipoproteins (HDL) can occur. (Ben-Hur et al., 1995; Costa-Tuna et al., 2024; Yslas et al., 2002) This can result in non-specific distribution and extravasation into different tissues, causing variability in biodistribution and reduced PDT effect in targeted neoplastic tissue. The extensive interaction with blood proteins can often lead to the recognition by the reticulo-endothelial system (RES), causing rapid blood clearance and extravasation into skin, liver, spleen and other tissues with higher prevalence of macrophages. Especially the accumulation in the liver can be particularly high, indicating its role in detoxifying the blood. (Nichols and Bae, 2012) Slowing down/minimizing the protein adsorption by enclosing the PS into micellar structures with non-fouling properties can resolve this issue and enhance the therapeutic effect (see illustration in Fig. 7). As an example, the micellar system designed for the indocyanine green delivery is described below. ICG is a negatively charged, near-infrared absorbing tricarbocyanine dye typically used as a photodynamic or photothermal agent with a very short lifetime of about 2-4 min, and limited extravascular distribution. Mundra et al. tried to resolve these issues, as well as ICG's binding to HDL_3_ lipoprotein, albumin and its extensive hepatobiliary secretion, by covalently conjugating amino-derivative of ICG to pendant carboxylic group of poly(ethylene glycol)-*b*-poly(2-methyl-2-carboxyl-propylene carbonate) (PEG-PCC) copolymer. They proved an increase in systemic circulation and enhanced tumor accumulation by whole body in vivo near infrared (NIR) imaging, observing a 2.5-fold higher mean fluorescence intensity of ICG conjugated micelles in tumor tissue at 24 h compared to ICG solution alone (p < < 0.001). Moreover, tumor signal intensity at 24 h was almost twice that of the rest of the body. (Mundra et al., 2015).

Indeed, encapsulating/conjugating PS into micellar drug delivery systems can prevent degradation (Chen et al., 2020) and increase the stability of otherwise labile PS, such as bateriochlorins (Pucelik et al., 2016a). It can also help prolonging the circulation in blood and increasing the uptake and internalization within the cancerous tissue. Indeed, undisputed benefit of micellar carrier is their ability to alter pharmacokinetics of PS. Variables such as the composition and overall architecture of the carrier (hydrophobicity/hydrophilicity, crosslinking, degradability, etc.) can be tuned to influence the absorption, distribution, metabolism and excretion. (Gibot et al., 2020; Liu et al., 2020; Mehraban and Freeman, 2015) Better accumulation inside the tumor can, in turn, minimize side effects on healthy tissues. Skin photosensitivity is a commonly encountered problem in PDT treatment. However, with PS encapsulation into micellar carrier and proper PDT timing, photosensitivity can be avoided. (Shieh et al., 2010).

The increased accumulation within tumorous tissue is typically assigned to the enhanced permeability and retention (EPR) effect - a passive targeting approach (see Fig. 7), relying solely on the increased permeability of macromolecules due to aberrant fenestration of tumorous blood vessels, and poor lymphatic drainage resulting in longer retention. (Matsumura and Maeda, 1986) For more than 40 years, the EPR effect has been used as one of the fundamental principles of cancer drug delivery, promising safe, simple and effective therapy. However, a closer look reveals, that animal models used to study the EPR effect differ from those used in clinical settings, making the EPR effect more pronounced in animals than in human patients. Indeed, Matsumoto et al. recently observed pulses in drug transfer, which showed that the EPR might not be constant in time, rendering the behavior even more complex (Matsumoto et al., 2016). Drug delivery in cancer patients is indeed much more difficult, with challenges such as irregular vascular distribution, poor blood flow inside the tumor and high tumor interstitial fluid pressure (IFP), the latter being a direct consequence of the EPR principle itself. The leaky vasculature transmits not only nanoparticles, but also the mix of proteins and other components in blood plasma, which are normally held by the walls of healthy blood vessels, maintaining osmotic gradient between the tumorous interstitial space and the capillary. In tumors, the osmotic gradient diminishes and the IFP begins to approach the microvascular pressure, meaning that the nanoparticles cannot be “pushed” inside the tumor as easily. (Nichols and Bae, 2014) Moreover, the tumor vasculature, as one of the major factors influencing the EPR effect, differs vastly from patient to patient, and tumor to tumor in crucial characteristics, like vessel density, perfusion, maturity and pore cutoff size. (Ojha et al., 2017) Strategies such as vessel permeabilization, normalization, disruption or promotion, including the administration of vasomodulators or vascular disrupting agents, promoting angiogenesis, or inhibiting the elevated levels of vascular endothelial growth factor (VEGF), are being developed. Regardless of the underlying mechanism, and however paradoxically they may sound, all the strategies aim to overcome the heterogeneity of the EPR effect and to enhance the tumor targeting. (Nichols and Bae, 2014; Ojha et al., 2017).

Another type of tumor targeting, so-called active targeting (see Fig. 7), relies on the incorporation of precise recognition motifs, aiming to specifically bind to cell surface or matrix molecules within the tumor. As opposed to passive drug targeting, relying solely on the pathophysiological properties of target tissue, the active targeting exploits highly (over)-expressed receptors by (i) cancer cells themselves (e.g. epidermal growth factor receptor (EGFR), transferrin receptor, folate receptor, prostate-specific membrane antigen (PSMA) or glycoproteins) or (ii) tumor endothelial cells (e.g. VEGF receptors VEGFR1 and VEGFR2, αvβ_3_ integrins or matrix metalloproteinases (MMPs)). (Danhier et al., 2010) The advantage of the latter lies in direct accessibility from the bloodstream without the need for drug extravasation (Böhmová and Pola, 2016).

Having a polymeric micellar carrier with various functional groups allows for its decoration with ligands to further enhance the specific delivery. Potara et al. covalently conjugated folic acid (FA) to chitosan covered IR780- loaded Pluronic F127 nanocarriers and evaluated their active targeting capacity against folate receptor expressing ovarian cancer cells (NIH-OVCAR-3) using both non-targeted carriers and folate-negative ovarian cancer cells (A2780-Cis) as controls. Both free IR780 and nanocarriers presented NIR fluorescence emission after only 30 min of incubation, indicating successful internalization. FA-functionalized carriers Plu-IR780-chit-FA exhibited visible more intense emission compared to non-targeted Plu-IR780-chit under the same conditions, using NIH-OVCAR-3 cancer cells. In case of comparison of folate-positive and folate-negative ovarian cancer cells, the NIH-OVCAR-3 cancer cells exhibited massive internalization of FA-decorated carrier, indicated by strong NIR fluorescence emission after only 10 min of incubation. Contrarily, very low fluorescence emission signals were detected for folate negative A2780-Cis cells, proving the increased specific internalization via folate receptor mediated uptake. (Potara et al., 2021).

**Fig. 8:**
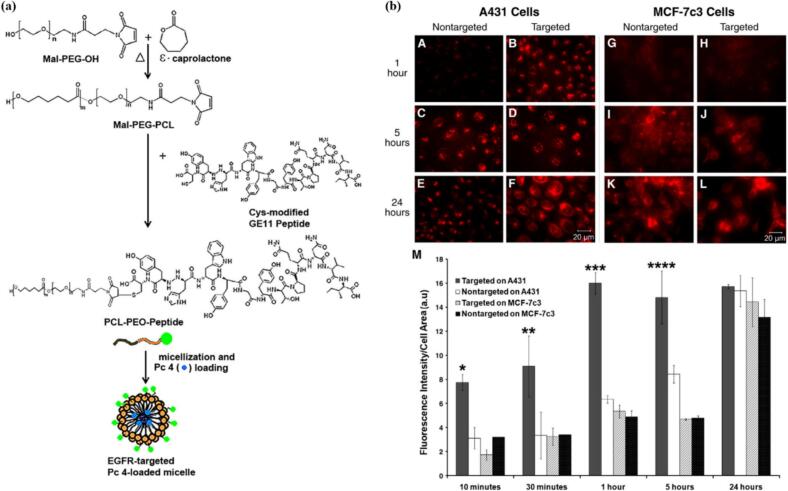
(a) Scheme for synthesis of Ma1-PEG-PCL, Cys-modified GE11 peptide conjugation to Ma1 termini via thioether linkage and Pc4 loaded GE11 decorated micellar nanoformulations; (b) representative fluorescence images comparing the Pc4 uptake at various timpoints when delviered via the EGFR-targeted vs. nontargeted nanoformluation on A431 cells (A-F) and MCF7-7c3 cells (G-L), the plot (M) shows quantitative Pc4 fluorescence intensity per cell area for various incubation time periods in these cells for the targeted and nontargeted nanoformulations. All data are for incubations with nanoformulation containing 400 nM Pc4. All levels of significance are for *P* < 0.05. Reproduced with permission from (Master et al., 2012). Copyright © 2012 Elsevier Inc.

Despite several other reports (Chang et al., 2018; Dou et al., 2018; Wang et al., 2018b) postulating a clear benefit of active vs. passive targeting in vitro, others are more skeptical, suggesting that the attachment of targeting moieties to micellar carriers leads only to faster internalization, but the active vs. passive uptake becomes similar (Master et al., 2012)/ or worse (Kunjachan et al., 2014) in the end. Mater et al. have synthesized actively targeted surface-modified silicon phthalocyanine (Pc4)-bearing PEG-PCL micelles by attaching 12-amino acid EGFR-targeting peptide, GE11, (Fig. 8 (a)), and evaluated the relative uptake of the EGFR-targeted (GE11-modified) and non-targeted unmodified micelles by EGFR-overexpressing A431 and EGFR-deficient MCF-7 cells. At the shortest incubation time studied (1 h), the actively targeted micelles delivered more Pc4 to the A431 cells than untargeted micelles did, whereas neither formulation delivered a detectable amount of Pc4 to the EGFR-deficient MCF-7 cells, suggesting a role of receptor mediated internalization. After 24 h, because of the long incubation period, both the membrane-mediated passive uptake via e.g. fusion or pinocytosis (which are well known time-dependent delayed process) and the receptor-mediated active uptake became similar in extent (Fig. 8 (b)) Their research supports the hypothesis, that micellar carriers with active targeting lead to faster (but not better), internalization and PDT effect, thus possibly providing efficient ways to reduce the drug-light interval (DLI). (Master et al., 2012) Importantly, the DLI itself plays crucial role and should be carefully considered when designing PDT protocols. By adjusting the delay between PS administration and light irradiation, it is possible to influence the predominant mechanism of action – determining whether the treatment primarily targets the vasculature (DLI ∼ 15 min) or tumor cells directly (DLI usually ∼24 h). (Repetowski et al., 2024).

Designing micellar carrier based on stimuli-responsive materials, or introducing a specific stimuli-responsive covalent bond in between the carrier and the active molecule, can ensure safe circulation in the blood without altering the photophysical properties, and also allow for triggered release of the PS in a slow and controlled manner, leading to improved phototoxicity over time. (Ding et al., 2011a) In addition, the intracellular localization of PS is important in determining PDT efficacy. Since ROS have a short half-life, their site of generation directly influences therapeutic outcomes and mode of cellular death. The PS's localization is largerly dependent on its chemical nature (f.e. molecular weight, lipophilicity, ionic charge, amphiphilicity). When a carrier is used, however, it may take over the pathway of cellular uptake. By incorporating a degradable and stimuli-responsive segment, it is then possible to enhance the accumulation in tumor, without interfering with the subcellular localization process of PS. (Castano et al., 2004) After fulfilling their role, the polymer carriers either remain in the tissue after cellular death, or they undergo exocytosis, return to the bloodstream and are eliminated via kidneys. The process of glomerular fitration strongly depend on the molecular weight, size and shape. (Markovsky et al., 2012) Majority of PS used are excreted via liver and bile. (Castano et al., 2005b) For more detailed information on the biological fate of the carrier and PS, we refer the reader to the dedicated reviews on such topics.

It is important to note, that the benefits mentioned here are not exclusive to polymeric micelles, as other nanocarrier systems also provide distinct advantages and face their own limitations in PS's delivery for effective PDT outcome. Each system offers unique physicochemical properties, drug loading capacities, and release profiles that may be advantageous in specific therapeutic contexts. (Luo et al., 2024).

### 2.5 Photosensitizer release mechanisms

**Fig. 9:**
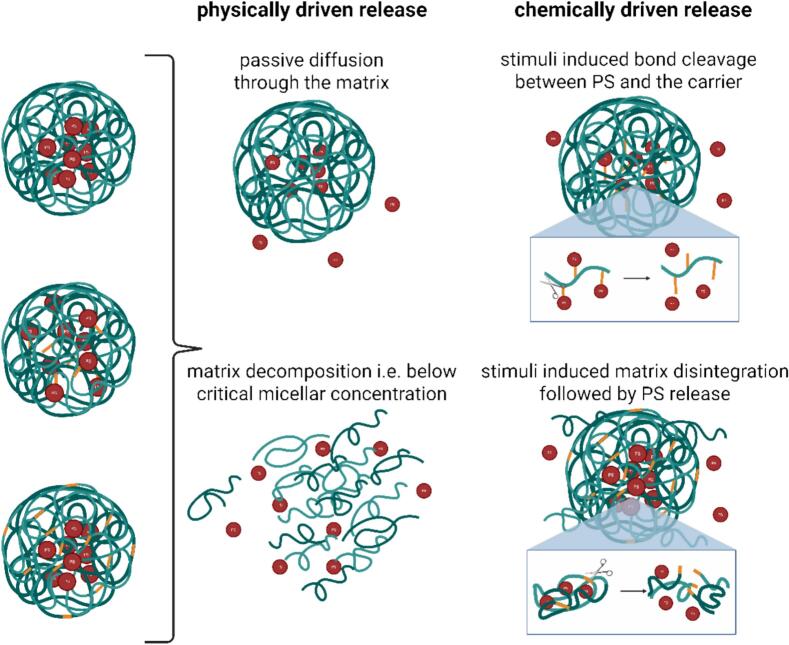
Possible mechanisms of photosensitizer release.

Depending on the selection of materials, different ways of releasing the PS can be achieved. In the following paragraphs, we will discuss non-triggered release based on simple diffusion or matrix degradation, as well as triggered release via various internal/external stimuli, leading to the disintegration of micelle or the disruption of covalent bonds between the PS and the polymer backbone. A summary of the various mechanisms and strategies for PS release from polymeric micelles, including both triggered and non-triggered approaches, is provided in Table 1, as well as an illustration of possible processes in Fig. 9.

**Table 1:** Various mechanisms of non-triggered and triggered release of photosensitizers.

Trigger	Mechanism	Ref.
**Non-triggered release**		
Passive release	Diffusion based release from hydrophobic core or slow degradation of polymer matrix	(Gibot et al., 2020; Van Butsele et al., 2009)

**Triggered release**		
pH	Acidic tumor/endosome environment causes polymer protonation, micelle destabilization, or linker cleavage	(Koo et al., 2010; Li et al., 2018b)
Redox	Intracellular glutathione (GSH) cleaves disulfide bonds or other redox-sensitive linkers	(Feng et al., 2019; Zhou et al., 2016)
ROS	Elevated oxidative stress leads to cleavage of ROS-sensitive spacers (i.e. thioether, thioketal/thioacetal…); Photosensitizer loaded carriers may act as self-triggering delivery systems	(Li et al., 2017b)
Temperature	Thermo-sensitive polymers undergo hydrophilic to hydrophobic phase transitions above LCST or below UCST; drug release or enhanced uptake can be achieved	(Huang et al., 2016)
Light	Light-induced molecular changes i.e. photoisomerization or photocleavage of spiropyran, o-nitrobenyl or coumarin photosensitive groups	(Trigo-Gutierrez et al., 2023; Xu et al., 2015b)
Enzyme	Cleavage of peptide linkers or polymer backbone by tumor-overexpressed enzymes (e.g. oxidoreductases, hydrolases, …)	(Han et al., 2015; Říhová et al., 1988; Yao et al., 2020)

#### 2.5.1 Non-triggered release

Non-triggered release in micellar delivery systems refers to passive and uncontrolled release of the usually physically encapsulated photosensitizer, without specific internal or external stimuli. This type of release is typically governed by the properties of the micelle's materials, resulting in diffusion of the PS through the matrix or the slow degradation of the micelles over time, under physiological conditions. (Gjuroski et al., 2019; Rodriguez et al., 2008) L. Gibot and her coworkers encapsulated pheophorbide a (Pheo) into three different amphiphilic block copolymers, namely poly(ethyleneoxide-*b*-ɛ-caprolactone) (PEO-PCL), poly(ethyleneoxide-*b*-D,l-lactide) (PEO-PDLLA) and poly(ethyleneoxide-*b*-styrene) (PEO-PS) and evaluated these systems based on their stability, release rates and in vitro efficacy. They linked the release of Pheo via diffusion with the affinity constants^2^ (*K*) between the PS and each type of polymeric micelles. PEO-PS exhibited the strongest affinity, resulting from the preferential location of Pheo within the hydrophobic core enhanced by the π- stacking between the PS's aromatic units with the porphyrin cycle of Pheo. As a result, PEO-PS exhibited the slowest release of Pheo from the micellar carrier with only 30 % in 4 days compared to about 50 % in three days in case of PEO-PCL. PEO-PDLLA micelles exhibited almost the same dialysis patterns as free Pheo, therefore this system was labeled as unsuitable for Pheo delivery. (Gibot et al., 2020; Gibot et al., 2014) The unfavorable release profile of PEO-PDLLA observed by Gibot et al. can be linked to the hydrolytic degradability of poly(α-hydroxy esters) such as PLA, PGA and PLGA.

Poor release performances (e.g. burst release) is typically linked with the encapsulation methodology and can lead to the immediate release of the majority of medication, causing increased side effects and lower effectiveness and bioavailability of the micellar delivery systems. However, the burst effect can be viewed from two perspectives. It is often seen as a negative consequence of the delivery system's design, that is unpredictable and cannot be significantly controlled. In applications such as wound healing, however, the immediate release of the load can be favorable. For targeting application with long circulation to increase the tumor accumulation, zero burst release remains desirable. (Huang and Brazel, 2001) Strategies such as core or shell cross-linking may stabilize the micellar carrier and decrease/ prevent the drug burst release during blood circulation. (Dantas et al., 2022; Talelli et al., 2015).

#### 2.5.2 Triggered release

Introduction of stimuli responsive linkages into the micellar carrier or between the polymer backbone and the photosensitizer can ensure that the active molecule is liberated in its active form only when and where it is needed. The triggered release can enhance the therapeutic efficacy while minimizing systemic toxicity. PS release from polymeric micelle systems can be precisely controlled through a number of internal and external stimuli, each offering distinct mechanisms for triggering micelle disassembly or the cleavage of covalently bound PS molecules. In addition, the choice of stimulus can influence the site of the release, intracellular trafficking and the resulting mode of cell death.

##### 2.5.2.1 pH-triggered release

**Fig. 10:**
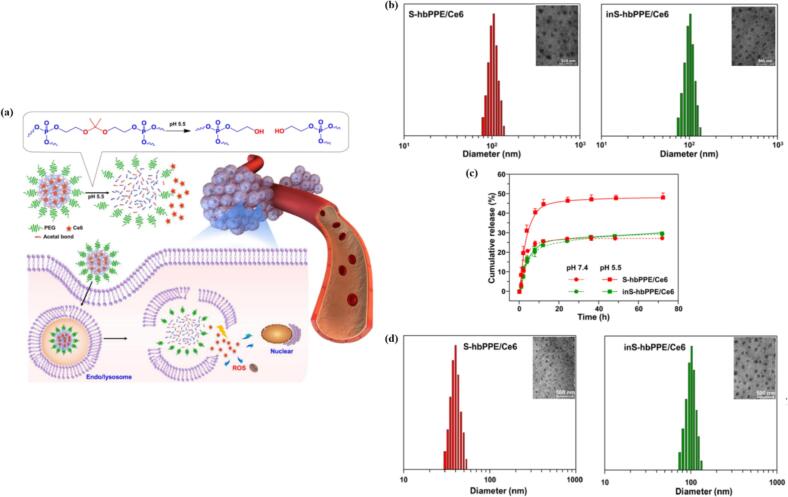
(a) Schematic illustration of a pH-sensitive acetal bond-linked hbPPE, S-hbPPE, as a nanocarrier of Ce6 (S-hbPPE/Ce6). The acetal bond linkers of S-hbPPE would be rapidly cleaved by the intracellular endo-/lysosomal acidic microenvironment, leading to triggered Ce6 release and enhanced ROS generation for PDT.; (b) Size distribution and TEM images in PBS, (c) release profiles of Ce6, and (d) size distribution and TEM images after incubation at pH 5.5 for 12 h, for pH-sensitive (S-hbPPE/Ce6) nanocarrier and pH-insensitive (inS-hbPPE/Ce6) control. Reproduced with permission from (Li et al., 2018a). Copyright © 2018 American Chemical Society.

One of the most commonly employed strategies for triggered release involves exploiting lower pH levels, taking advantage of the more acidic environments found in endocytic vesicles or tumor microenvironment compared to the normal tissue or cytoplasm. It is well documented that solid tumors have slightly acidic extracellular microenvironment, arising from the elevated glycolytic activity of cancer cells, which leads to the accumulation of lactic acid. The endocytic vesicles are even more acidic, exhibiting pH of 6.8-5.9 in early endosome, or pH 6.0–4.9 in the late endosome/lysosome. (Huotari and Helenius, 2011; Tafech and Stéphanou, 2024) Incorporation of pH-sensitive or responsive groups or linkages, such as hydrazone (Islam et al., 2023; Tavares et al., 2023), ketal (Malarz et al., 2025)/acetal (Li et al., 2018a) or ionizable groups (i.e. amines) (Dai et al., 2017; Koo et al., 2010; Wang et al., 2016) can lead to the cargo release either by bond cleavage or by changing the solution properties of the carrier. Li et al. prepared a novel PEG-ylated hyperbranched polyphosphoester with encapsulated chlorin e6 (S-hbPPE/Ce6). These nanocarriers contained pH-sensitive acetal linkages, which readily cleaved upon exposure to the *endo*-/ lysosomal acidic environment. This disassembly of the carrier led to increased release of Ce6 and subsequent shrinkage in size, which was not observed for pH-insensitive control (inS-hbPPE/Ce6) (Fig. 10). (Li et al., 2018a) In parallel, polymers with ionizable groups can undergo physical changes in response to pH shifts. Koo et al. synthesized pH-responsive MPEG poly(β-amino ester) polymeric micelles for the encapsulation of PpIX. The micelles showed sharp pH-dependent de-micellization at acidic extracellular tumorous pH due to the protonation of tertiary amine groups and resulting in a shift from hydrophobic to hydrophilic amino ester block. (Koo et al., 2010).

Indeed, given that majority of delivery systems are internalized via the endocytosis pathway, endosomal entrapment is commonly encountered. Due to the presence of weakly basic functionalities such as amines, which can be protonated and sequester protons from the surrounding medium, certain materials can buffer the acidic pH within endo- / lysosomes, causing osmotic swelling and rupture, with subsequent release of encapsulated cargo into the cytoplasm. This effect, known as the proton sponge effect is a powerful tool for further re-localization of released cargo to other intracellular compartments. (Dai et al., 2017; Desai et al., 2024).

##### 2.5.2.2 Redox-triggered release

Redox sensitive delivery systems have emerged as powerful tools for enhancing the selective release of photosensitizers within tumorous cells. These systems exploit the distinctive redox conditions of intracellular environment, especially the elevated levels of glutathione (GSH) found in the cytosol, compared to extracellular regions. (Cheng et al., 2011) The most common approach in redox-responsive PDT systems involves the use of disulfide bonds (S—S), which are readily cleaved by GSH (Gao and Lo, 2018; He et al., 2023; Liu et al., 2015; Zhou et al., 2016). Zhou et al. synthesized reduction-responsive porphyrin monomer bearing a disulfide bond (TPPC6SAM) and used it to construct a series of block copolymers with oligo(ethylene glycol) methyl ether methacrylate (OEGMA), POEGMA-*b*-PTPPC6SAM with varying lengths of PS-containing block. Regardless of the block length, they all exhibited more than 80 % of photosensitizer release after 24 h exposure to 10 mM GSH as well as changes of the carrier sizes measured by DLS. (Zhou et al., 2016) Similar results – increased cargo release upon exposure to 10 mmol L^-1^ GSH, supported with complete collapse of the delivery system observed by transmission electron microscope (TEM), was achieved by He et al. using disulfide containing MPEG-SS-PMLA@Ru complex micelles. (He et al., 2023) In addition to disulfide-based systems, more advanced designs incorporate selenide (Se—Se) (Feng et al., 2019), which is even more sensitive to redox conditions due to their weaker bond energy. These newer linkers respond more rapidly or at lower GSH concentrations, providing faster or more finely tuned release kinetics. However, due to their potential cytotoxicity resulting in selenosis in cells, more organic counterparts like tellurium can be used. (Fan et al., 2016; Sikder et al., 2022).

Importantly, there is evidence suggesting that GSH functions as a scavenger of reactive oxygen species. By introducing GSH-sensitive linkers into the polymeric carrier, it may be therefore possible to deplete GSH, elevating ROS levels and enhancing PDT efficacy. (Liang et al., 2018; Wang et al., 2022; Xia et al., 2021).

##### 2.5.2.3 ROS-triggered release

**Fig. 11:**
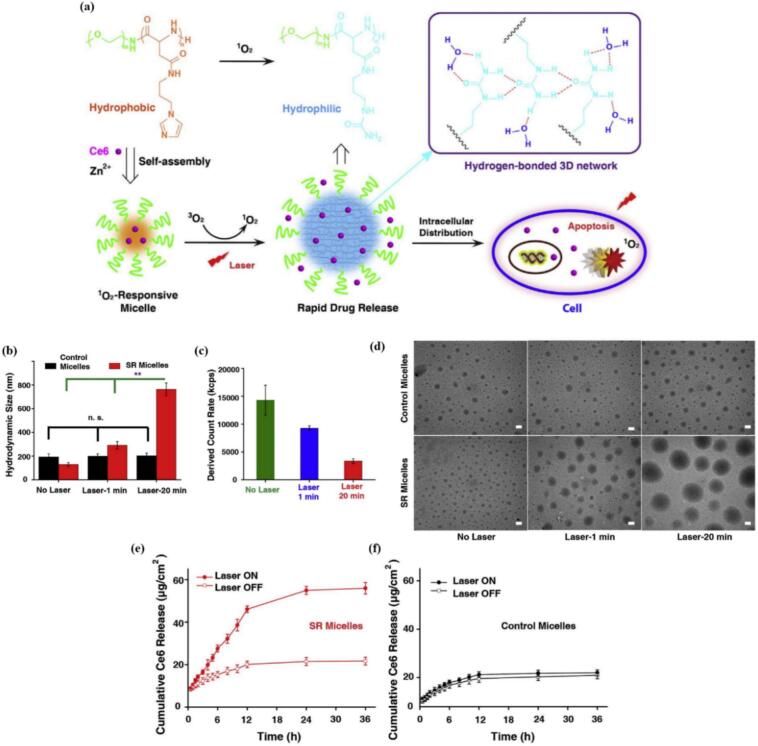
(a) illustration of singlet‑oxygen responsive micelles for enhanced photodynamic therapy. The imidazole-bearing amphiphilic copolymer can self-assemble into micelles containing Zn^2+^ crosslinkers. Chlorin e6 (Ce6) was encapsulated within micelles. Laser activation generates singlet oxygen, thereby converting imidazole to urea, and resulting in micelle expansion, rapid Ce6 release, and onset of apoptosis. Upon size increase, the urea-containing polymers form various types of hydrogen bonds and do not lose nanocarrier integrity.; (b) hydrodynamic size of singlet oxygen-resposnive (SR) and control micelles without and with laser treatment (660 nm, 100 mW/cm^2^). “n.s.” indicates no statistical difference, ** indicates *P* < 0.01; (c) derived count rate of micellar samples by dynamic light scattering upon laser treatment (1 min, 20 min), and the attenuation factor was 0.0126 (no laser), 0.0126 (1 min), and 0.044 (20 min), respectively (automatic mode); (d) transmission electron microscope images of both micelles (scale bar: 200 nm).; (e) cumulative Ce6 release from SR micelles. “Laser ON” indicates the conditions of laser treatment for 10 min; (f) cumulative Ce6 release from control micelles. Reproduced with permission from (Li et al., 2017b). Copyrights © 2017 Elsevier B.V.

ROS-triggered release is an interesting sub-type of redox-triggered release. While high ROS levels can induce cellular death, it has also been observed that slightly increased ROS can promote DNA mutations, cell proliferation and migration. As a result, cancer cells have developed mechanisms to tolerate slightly increased levels or ROS/ oxidative stress to support their growth. ROS are therefore a double-edged sword and can also be employed as trigger stimulus. (Pelicano et al., 2004) Linkers/groups like thioether, aryl boronic esters, peroxalate esters, thioketal/thioacetal and many others are known to be selectively sensitive towards various ROS. (Zhao et al., 2021) In the realm of PDT, this approach is particularly interesting, as the PS-loaded nanocarriers can act as self-triggered delivery systems via either (i) cleavage of the covalently linked cargo or (ii) solubilization and degradation of delivery vehicle followed by cargo release. Li et al. synthesized biocompatible Zn^2+^-crosslinked methoxyl poly(ethylene gly*co*l)-*co*-poly(aspartic acid) with conjugated imidazole as smart on-demand PDT nano-system. The stable and tight coordination complexes between Zn^2+^ and imidazole would stabilize the micelles during circulation, but upon ^1^O_2_ exposure, the coordination complex is distrupted, accompanied by transformation of imidazole to urea and increased water solubility. Such transformation leads to significant size expansion followed by release of loaded Ce6 as photosensitizer (see Fig. 11). (Li et al., 2017b).

##### 2.5.2.4 Temperature-triggered release

Polymers, such as PNIPAM, poly(*N*-vinylisobutyramide), poly(2-isopropyl-2-oxazoline) or Pluronics (PF-127) are well known materials with thermo-responsive behavior. These polymers change their conformation or physicochemical properties across a phase transition temperature, known as the lower critical solution temperature (LCST). LCST-type thermo-responsive polymers are highly hydrated and in an extended random-coil structure below the transition temperature, whilst collapsing into hydrophobic globules above the temperature due to dehydration. (Akimoto et al., 2014; Kim et al., 2010) PNIPAM, as the most exploited material, undergoes phase separation upon heating in aqueous solution above 32 °C. Despite this temperature being close to that of human body, PNIPAM alone may not be suitable for in vivo applications, as it would rapidly aggregate upon injection. (Talelli and Hennink, 2011) Copolymerization with other comonomers is crucial for altering the LCST, since molecular weight and polydispersity alone has only minimal effect. (Throat and Bhattacharya, 2024b) The thermo-responsive materials can be used as either core- or corona-forming segment of the block copolymer. The drug release can then be achieved by induced hyper- or hypo-thermia. (Akimoto et al., 2014; Appold et al., 2017; Nakayama et al., 2006; Talelli and Hennink, 2011).

**Fig. 12:**
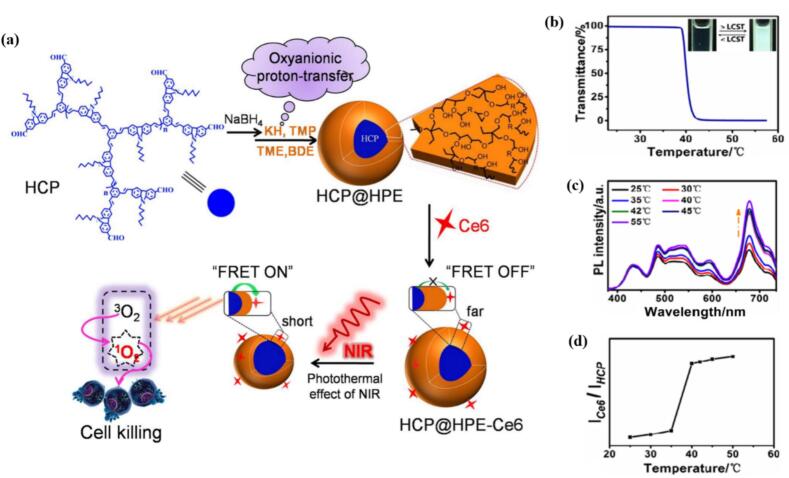
(a) synthesis of thermoresponsive HCP@HPE unimolecular micelles and illustration of the combination of 2P-FRET and photothermal effect of NIR for photodynamic therapy.; (b) Temperature dependence of optical transmittance at 550 nm for HCP@HPE 3 aqueous solution. Inset: Digital photograph of HCP@HPE 3 aqueous solution when heating and cooling. (c) Normalized PL spectra of HCP@HPE 3-Ce6 aqueous solution with variable temperature at 25, 30, 35, 40, 42, 45, and 55 °C. (d) ICe6 /IHCP values from HCP@HPE 3-Ce6 as a function of temperature. Reproduced with permission from (Huang et al., 2016). Copyright © 2016 American Chemical Society.

Beyond triggering release, thermo-responsive materials can also enhance cargo uptake. Due to the collapse and aggregation of polymer micelles upon heating above LCST, the hydrodynamic radius increases, resulting in enhanced passive accumulation inside the tumorous tissue. (Akimoto et al., 2009; Yamada et al., 2024) For further spatial and temporal control over the responsiveness, external stimuli such as high-intensity focus ultrasound (HIFU) for precise local heating (Akimoto et al., 2014) or near-infrared irradiation can be employed. Huang et al. developed core-shell unimolecular hyperbranched micelles composed of two-photon-absorption (2PA) hyperbranched conjugated polymer (HCP) core and thermo-responsive hyperbranched polyether (HPE) shell, named HCP@HPE. Upon NIR-irradiation-induced heating above the polymer's LCST (39.1 ± 0.1 °C), the thermo-responsive shell collapsed, shortening the space between the HCP and Ce6 grafted on the surface. By shortening the distance between HCP and Ce6, the fluorescence resonance energy transfer (FRET) was switched “on”, providing remarkable enhancement of tumor inhibition both in vitro and in vivo. Most importantly, the ^1^O_2_ production by HCP@HPE-Ce6 micelles under irradiation at 800 nm via the 2P-FRET strategy was much higher than that of free Ce6 treatment under traditional 650 nm laser irradiation (Fig. 12). (Huang et al., 2016).

Second type of thermo-responsive materials possess upper critical solution temperature (UCST). Upon temperature increase, they undergo hydrophobic to hydrophilic transition resulting in disassembly of polymeric micelles into individual unimolecular chains, accompanied by drug release. Although they have not been widely used until recently, the UCST-type thermo-responsivity is gaining adequate attention as drug carriers (Bordat et al., 2019; Zhang et al., 2014), including for PDT (Jiang et al., 2019; Ji et al., 2022).

##### 2.5.2.5 Light-triggered release

**Fig. 13:**
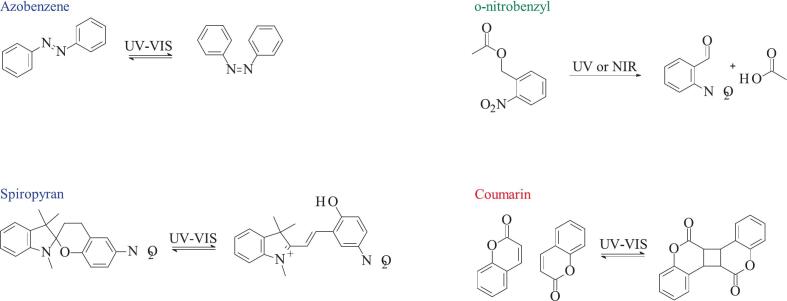
Examples of light-triggered transformations. Replicated from (Kathuria and Kumar, 2025).

Light can be used not only to activate the photosensitizer but also as a precise external stimulus for controlled cargo release from the delivery system. However, there is a frequent simplification (or perhaps semantic ambiguity) in the field: many systems are often described as “light triggered”, when in fact the actual release mechanism is mediated e.g. by ROS generated upon light exposure. In these cases, light merely serves as an external means to initiate ROS production, which then induces physical or chemical changes in the carrier system. (Liu et al., 2018) In contrast, in “truly” light-triggered systems, light itself directly drives molecular changes – typically via photoisomerization or photocleavage – that lead to cargo release. Photosensitive groups, such as azobenzene or spiropyran undergo light-induced isomerization, while o-nitrobenzyl or coumarin are cleaved or fused upon irradiation, respectively (Fig. 13). These changes may or may not be reversible. (Kathuria and Kumar, 2025; Rwei et al., 2015) Much of the current research focuses on designing photo-responsive micellar carriers for the delivery of anticancer agents or model hydrophobic drugs. (Cheng et al., 2019; Jiang et al., 2007; Jin et al., 2011; Lee et al., 2014; Pearson et al., 2015; Wang et al., 2018a).

**Fig. 14:**
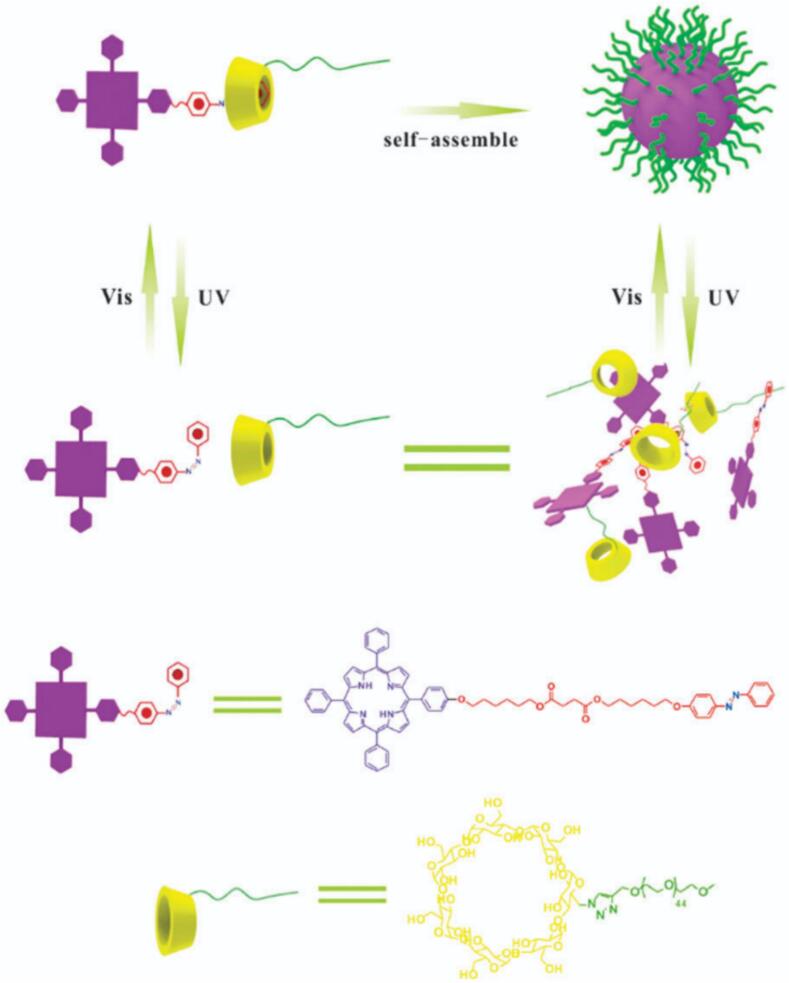
Self-assembly and disassembly of TPP–Azo/PEG–b-CD host-guest supramolecular amphiphiles. Reproduced with permission from (Xu et al., 2015b). Copyrights © 2015 The Royal Society of Chemistry.

Nonetheless, there are several promising studies demonstrating the applicability of light-responsive systems in PDT as well. Trigo-Gutierrez et al. successfully encapsulated curcumin (CUR) into photo-responsive poly(ethylene glycol) conjugated with 4-bromomethyl-3-nitrobenzoic (BNA) group followed by grafting of phenoxyacetic acid (PAA), which were able to self-assemble into micelles intended for antimicrobial PDT (aPDT). Compared to light-non-responsive systems based on Pluronics, the light-responsive micelles exhibited faster CUR release upon irradiation (λ = 355 nm), were non-toxic to mammal cells as well as caused inactivation of several pathogenic microorganisms. (Trigo-Gutierrez et al., 2023) In another study, Xu et al. developed smart, light-responsive supramolecular system for controlled PS release and enhanced PDT. The micelles were formed via host-guest interactions between a porphyrin-azobenzene conjugate (TPP-Azo) and poly(ethylene glycol)-β-cyclodextrin (PEG-β-CD), self-assembling into well-defined structures. The *trans*-Azo isomer matches well with β-CD via hydrophobic and van der Waals interaction force, while the *cis*-Azo isomer escapes from the β-CD cavity, leading to separation and release of TPP upon light exposure at 450 nm (Fig. 14). (Xu et al., 2015b).

Despite significant advances in the development of photosensitive carriers, certain limitations remain. Especially, the need for irradiation within the UV/VIS range may be problematic for biomedical applications due to the rather limited penetration into living tissues or potential damages by such wavelengths. (Gohy and Zhao, 2013) Limitations will be discussed later on in more detail.

##### 2.5.2.6 Enzyme-triggered release

In tumor cells, enzymes from nearly all seven major classes (McDonald and Tipton, 2023) may be overexpressed, each contributing to key processes supporting malignancy, and they can be strategically exploited to trigger the controlled release of the photosensitizer from polymer micelles. Among these classes, oxidoreductases are the most widely studied due to their “simple” action mechanism, involving the transferring of an electron or hydrogen atom from one molecule to another.

###### 2.5.2.6.1 Oxidoreductases

Tumorous tissues are characterized by a more reducing environment compared to normal tissues due to the increased production of NADH resulting from the switch from oxidative phosphorylation to glycolysis. The imbalance between NAD+ and NADH changes the cellular redox potential and favors the overexpression of many redox enzymes. (Liao et al., 2023) Azobenzene moieties, discussed before for their *cis*-*trans* isomerization, can be reduced to aniline under hypoxic conditions. Incorporating them into micellar carriers of PSs can help with their release even under hypoxic conditions. (Li et al., 2018a; Xu et al., 2020).

**Fig. 15:**
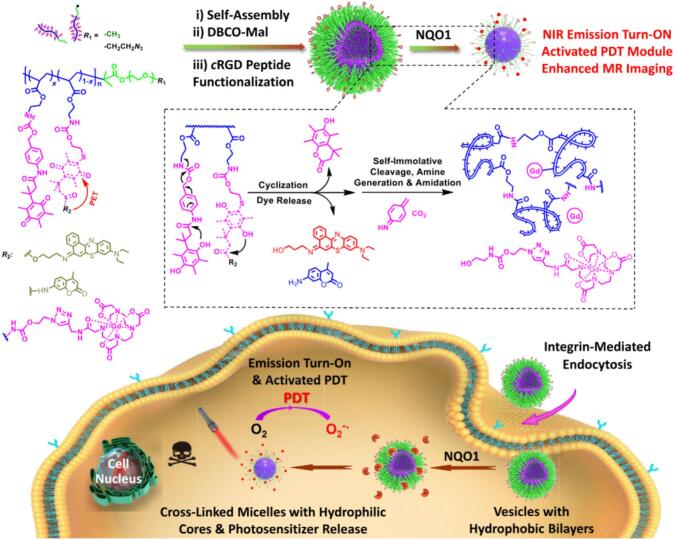
Tumor-cell-targeted NQO1 enzyme-responsive polymeric vesicles were fabricated via self-assembly of amphiphilic BCPs containing quinone trimethyl lock-capped self-immolative side linkages and quinone-bridged photosensitizers (coumarin and Nile blue) in the hydrophobic block. Initially, fluorescence emission and photodynamic therapy (PDT) potency are in the “off” state due to aggregation-caused quenching and quinone-rendered PET quenching. Upon cellular uptake, the cytosolic NQO1 enzyme triggers extensive decaging reactions of side linkages and fluorogenic release of conjugated photosensitizers, leading to “turn-on” of fluorescence imaging and PDT modules. This process is accompanied by the transformation of vesicles into cross-linked micelles with smaller sizes and hydrophilic cores, which could be directly monitored via enhanced magnetic resonance (MR) imaging signals. Reproduced with permission from (Yao et al., 2020). Copyright © 2020 American Chemical Society. (For interpretation of the references to colour in this figure legend, the reader is referred to the web version of this article.)

**Fig. 16:**
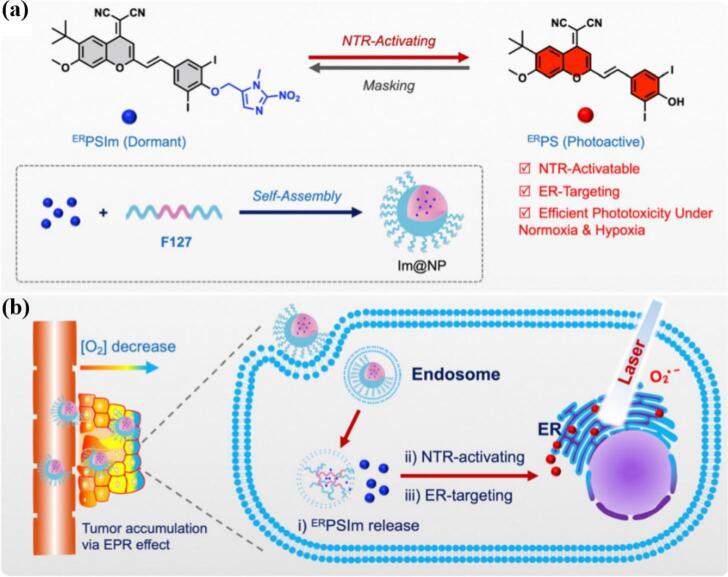
(a) Schematic illustration of NTR-activatable photosensitizer ^ER^PSIm and its nanoformulation Im@NP. (b) Utilization of Im@NP for hypoxia-activated and ER-targeted PDT against hypoxic solid tumors. Reproduced with permission from (Zhang et al., 2023a). Copyrights © 2023 Royal Society of Chemistry.

Among various investigated enzymes, NAD(*P*)H:quinone oxidoreductase isozyme 1 (NQO1) is upregulated in breast, pancreatic, colorectal, cervical and lung cancer and as such can serve as a trigger for precise delivery. Yao et al. developed self-assembled vesicles from an amphiphilic block copolymer containing quinone trimethyl lock-capped self-immolative side linkages and quinone-bridged photosensitizers, namely coumarin and Nile blue. Upon internalization, the NQO1 enzyme triggers cleavage of quinone linkages and fluorogenic release of conjugated PSs, leading to NIR fluorescence emission turn-on and activated PDT (Fig. 15). (Yao et al., 2020) Similarly, Zhang et al. designed hypoxia-activated Pluornic F127-encapsulated theranostic agent ^ER^PS with nitroaromatic spacers, namely *p*-nitrobenzyl moieties and 2-nitroimidazole-based groups. Under normal conditions, these groups block the activity of ^ER^PS, but under hypoxia, the intracellular nitroreductase reduces the nitro group to an amine, triggering cleavage of the spacer and releasing the PS in its active form (Fig. 16). (Zhang et al., 2023a).

###### 2.5.2.6.2 Hydrolases

Second, the most studied enzymes for triggered drug release are hydrolases. Micellar carriers based on hyaluronic acid (HA) encapsulating a PS can not only be passively accumulated by the EPR effect, but also via the CD44 HA-receptor mediated endocytosis. Additionally, the carrier can be disassembled through degradation of the HA backbone by hyaluronidase abundant in the cytosol of the tumor cells, ensuring both efficient targeting capabilities as well as sustained triggered release of the encapsulated photosensitizer. (Li et al., 2016; Yoon et al., 2012) Similarly, introducing carboxylate ester bonds into a micellar carrier with an encapsulated photosensitizer can render the system susceptible to carboxylesterase (CE). (Wang et al., 2023b).

Proteases such as MMPs or cathepsins are probably the most used hydrolases for targeting as well as enzymatic cleavage and micelle destabilization. MMPs, as endopeptidases, contribute to tumor cell proliferation, adhesion, and differentiation, but they are primarily known for degrading key components in the extracellular matrix. (Isaacson et al., 2017) Similarly, cysteine cathepsins also contribute to matrix proteins degradation thanks to their localization on cell membranes and secretion in endosomal or lysosomal vesicles. (Mohamed and Sloane, 2006).

Several studies highlight diverse strategies for exploiting MMP-sensitive delivery systems to enhance specificity and efficacy of PDT. They employ specific cleavable peptide linkers, surface charge reversal, PEG shedding, or payload release within the tumor, with PSs conjugated or encapsulated that remain inactive until enzymatic cleavage by MMPs activates or exposes them. These platforms integrate additional functionalities, such as pH sensitivity, β_2_-microglobulin-derived anti-phagocytic stealth coating or PD-L1 antibodies, demonstrating how MMP-responsive mechanisms can be tailored to improve tumor penetration, control the activation of PDT agents, and be combined with chemotherapy or immunotherapy. (Bao et al., 2022; Jia et al., 2022; Shu et al., 2021; Su et al., 2021) Han et al. developed a smart, dual-function probe called TPPP, enabling precise tumor detection and PDT. The probe links PpIX with an aggregation-induced emission (AIE) fluorophore, tetraphenylethylene (TPE) via PEGylated Pro-Leu-Gly-Val-Arg (PLGVR) peptide sequence as a linker. Upon MMP2-cleavage in the tumor microenvironment, TPE is released, aggregates, and emits strong fluorescence, while PpIX remains constant and serves both as a therapeutic ROS generator and a reference signal. This ratiometric system integrates tumor-triggered activation, real-time imaging and therapy in a single molecular platform. (Han et al., 2015).

Among cysteine proteases, cathepsin B is one of the most prominent and widely used enzyme for controlled and site-specific release of therapeutic payload. Prof. Kopeček and his research group were pioneers in the development of the GFLG peptide linker, cleaved by cathepsin B. (Říhová et al., 1988) Their HPMA-Dox conjugate, known as PK1, progressed into clinical trials. (Vasey et al., 1999) The group also explored the application of the GFLG linker in photodynamic therapy, enabling targeted delivery of photosensitizers such as meso-chlorin e6 monoethylene diamine disodium salt (Mce6) (Krinick et al., 1992) and ZnPc (Gu et al., 1993). In recent years, Luo et al. have built upon the cathepsin B-cleavable GFLG linker by developing an advanced delivery system designed to achieve a synergistic effect between chemotherapy and two-photon PDT. Their platform, based on poly [oligo (ethylene glycol) methyl ether methacrylate] (polyOEGMA)-functionalized dendritic polymer-paclitaxel prodrug with a hydrophobic core was designed and prepared to encapsulate pyropheophorbide a (PPa) and an imidazole derivative, a two-photon absorption compound T1. The GFLG spacer was inserted into the branches of the dendritic polymer, and also used to bind paclitaxel (PTX). Upon exposure to cathepsin B, the cleavage would result in sustained release of PTX as well as partial degradation of the carrier. (Luo et al., 2021).

In contrast, even though other enzyme classes – transferases, lyases, isomerases, ligases, and translocases – are conceptually interesting, they are less frequently employed in micellar drug delivery compared to oxidoreductases and hydrolases. (Wang et al., 2021).

To sum up, a lot of interesting research has been conducted on enzyme-activatable molecular photosensitizers as well, offering highly selective imaging and therapeutic capabilities. The majority of these photosensitizers remain in their inactive “OFF” state, until triggered by a specific enzyme, such as alkaline phosphatase (Xiong et al., 2024; Zhang et al., 2023a), monoamine oxidase A (Hu et al., 2023), or carboxylesterase (Wang et al., 2023a). Together, these novel photosensitizers showcase the potential of enzyme-responsive systems for precise and effective cancer theragnostic.

## 3 Shortcomings and future perspective

Despite being a promising cancer treatment, photodynamic therapy still faces several key limitations that hinder its broader clinical applications. PDT can be effectively combined with other therapeutic approaches (whether conventional (Grin et al., 2022) or more sophisticated (Alvarez and Sevilla, 2024)) to enhance overall treatment efficacy and overcome its inherent limitations. In addition, different drug release strategies may be integrated to achieve synergistic effects, enabling more precise and efficient cancer therapy. (Obalola et al., 2025; Shu et al., 2021; Xu et al., 2020) Recently, combination of PDT with immunotherapy, particularily employing immune checkpoint inhibitors has gained a lot of attention, as it can synergistically enhance the anti-tumor response, and induce not only regression of primary tumors, but also distant metastases. (Warszyńska et al., 2023) Similarily, combination of PDT with PTT could create a synergistic effect to destroy cancer cells more effectively than either therapy alone. PDT can enhance the sensitivity of tumor cells to PTT by modulating the tumor microenvironment, while the heat produced by PTT improves oxygenation, enhances bloodflow and strengthens PDT efficacy. Moreover, phototherapy-induced tumor debris can act as tumor-associated antigens, triggering antitumor immune responses. (Kong and Chen, 2022) Multifunctional micellar system for combined PTT and PDT containing NIR dye was investigated to determine the influence of the multimodal imaging to guide the therapy to higher anticancer efficacy. (Gong et al., 2014).

One major challenge, which has already been discussed is hypoxia. Since PDT, and particularly the type II mechanism, relies on molecular oxygen, the efficacy of the treatment strongly depends on the oxygen supply. (Josefsen and Boyle, 2008b) Switching the mechanism from type II to type I, which involves hydrogen-atom abstraction or electron-transfer between the excited photosensitizer and a substrate, can amplify PDT response, particularly in case of low oxygen levels. (Josefsen and Boyle, 2008b; Zhang et al., 2023b) Ding et al. encapsulated mTHPP into two separate carriers, (i) electron-rich PEG-*b*-PDPA and (ii) electron-deficient PEG-*b*-PLA. They hypothesized, that the electron-rich micelle carrier can act as electron reservoir, promoting type I reaction – despite using a type II PS agent. Indeed, they observed positive effect of electron-donating PDPA segment on formation of superoxide radicals, able to compete with energy transfer process generating ^1^O_2_ under aerobic conditions. However, under hypoxic conditions, the electron transfer becomes dominant. Such findings indicate that polymeric micelles can also serve to modulate type I and/or type II photochemical reactions for effective ROS generation. (Ding et al., 2011b).

**Fig. 17:**
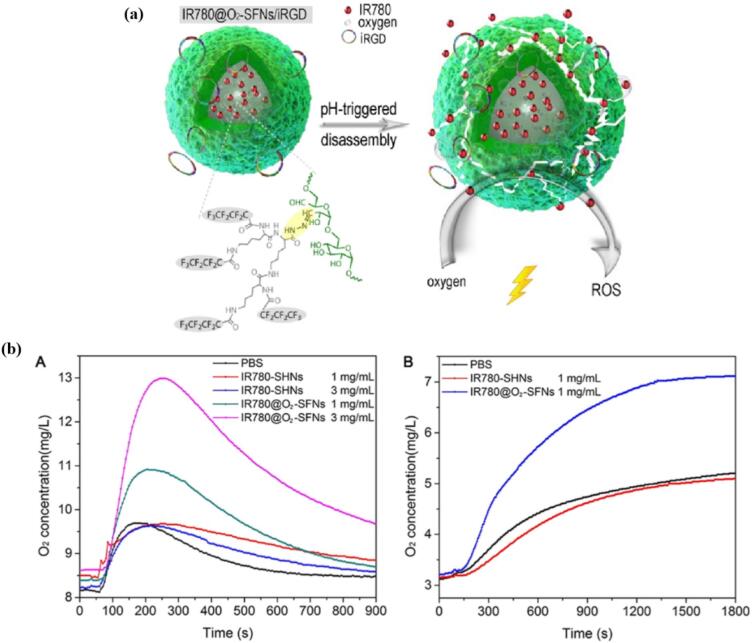
(a) Schematic illustration of an oxygen self-sufficient and tumor-penetrating nanoplatform for enhanced PDT. Structure and mechanism of IR780@O_2_-SFNs/iRGD nanoplatform. IR780 and oxygen are loaded in the core of SFNs. The cleavage of hydrazone bond in pH 5.0 results in the disassembly of nanoplatform. Accompanied by self-sufficient oxygen, IR780 induces ROS generation under NIR irradiation.; (b) Oxygen loading and release behavior. A: O_2_ concentration changes after addition of IR780@O_2_-SFNs, IR780-SHNs, and PBS into PBS buffer without deoxygenating. The samples were added into PBS at the 60 s. Also, the oxygen concentration was monitored all the time by a dissolved oxygen meter (DOG-3082). The process was conducted at 1 mg mL^-1^ and 3 mg mL^-1^ of the nanocarriers. B: Oxygen release behavior of IR780@O_2_ -SFNs, IR780-SHNs, and PBS in deoxygenated PBS buffer. The deoxygenated PBS was processed by nitrogen, which was also monitored by a dissolved oxygen meter (DOG-3082). Nanoplatforms (1 mg mL^-1^) were adopted to calculate the oxygen loading capability. Reproduced with permission from (Ma et al., 2019). Copyrights © 2019 American Chemical Society.

In addition to tumor-induced hypoxia, the photodynamic therapy process itself can further induce depletion of oxygen and potentiate hypoxia. To combat this oxygen consuming nature, developing strategies to effectively increase the oxygen levels in tumors is of great importance for PDT efficacy. To highlight several strategies, one can increase the blood flow to enhance the supply, or create an oxygen reservoir and delivery system. (Huang et al., 2021) Ma et al. designed IR780 loaded pH-sensitive fluorocarbon-functionalized nanoparticles with iRGD as a tumor targeting peptide (SFNs). Because of the high electronegativity of fluorine and high hydrophobicity of perfluorinated carbon molecules, the hydrophobic core of SFNs possessed excellent oxygen affinity and loading and as such can significantly relieve the tumor hypoxia (Fig. 17). (Ma et al., 2019) Indeed, the positive effect of fluorination on photophysical, photochemical and electrochemical behavior of PS, or the polymeric carrier, was also observed by Pucelik et al. and Tseng et al., respectively. Not only do the fluorine atoms favor intersystem crossing due to heavy atom effect, consequently enhancing the ^1^O_2_ quantum yields, their known affinity to oxygen also helps increasing the amount of oxygen molecules surrounding the photosensitizer. (Pucelik et al., 2016b; Tseng et al., 2021) Overall, these respective researches indicate the potential of fluorination to improve the PDT efficacy in hypoxic conditions.

Another limitation lies in the restricted tissue penetration of light. Light can be absorbed by molecules like hemoglobin melanin and water, or scattered by the refractive index mismatch between cellular components, deflecting the photons away from a direct path and limiting the penetration depth. In addition, shorter wavelengths are absorbed and scattered more than longer wavelengths, leading to shallower penetration. PDT is therefore suitable mainly for the treatment of superficial tumors, requiring penetration of several nanometers. However, for deeper-seated or larger tumors (> 1 cm) more advanced PSs or light sources are required. Much interesting research has focused on the design and development of novel light delivery sources, ranging from laser optical devices, optical fibers, planar devices as well as injectable devices. (Algorri et al., 2021; Finlayson et al., 2022; Seung Lee et al., 2022) Other alterations to improve the penetration depth and thus the effectiveness of PDT can be made on the field of photosensitizers. The so called “biological window” allows for longer wavelengths to pass through. Using photosensitizers that absorb in this NIR region can lead to deeper penetration and more pronounced PDT effect in deep-seated tumors. In such a case, the usefulness of PDT for other types of tumors that are not achievable with classical PS is significantly increased. Much attention is focused on the use of chromophores such as porphycenes, chlorins, bacteriochlorin, phthalocyanines or structurally modified BODIPYs. (Breitenbach et al., 2019b; Chinna Ayya Swamy et al., 2020; Liu et al., 2016; Paul and Biswas, 2025).

In two-photon absorption photodynamic therapy (2PA-PDT), NIR light enables unique non-linear process in which two photons of longer wavelength are absorbed simultaneously by the photosensitizer instead of a single photon of higher energy. Compared to “conventional” PDT, it allows one to avoid the UV/VIS region and offers better spatial controlled, limited tissue photodamage, and minimal autofluorescence from background. (Shi et al., 2022) Unfortunately, photosensitizers used for 1P-PDT are not suitable for 2P-PDT, because they possess low values of 2PA cross sections (σ_2_). Various synthetic strategies, especially the introduction of conjugated systems, donor or acceptor moieties and enhancement of molecular coplanarity are employed to improve the σ_2_ values. By increasing the size of the π-electron system and the distance between the donor (HOMO) and acceptor (LUMO) moieties, large enhancements can be obtained. (Gierlich et al., 2022).

Last but not least, continued efforts in the synthesis and design of smarter, more efficient, and sophisticated delivery systems will be essential for further advancement of PDT. Indeed, several challenges must still be addressed before the carrier systems can achieve full clinical translation. Long-term safety and biodegradability remain critical considerations, as incomplete degradation or accumulation of polymer residues could present potential risks. Equally important is the issue of scalability — the transition from laboratory synthesis to reproducible and cost-effective manufacturing remains a major hurdle. Therefore, future research should not only focus on improving therapeutic efficacy but also ensure that polymeric nanocarriers meet the requirements of safety, sustainability, and large-scale production. (Ezike et al., 2023; Markovsky et al., 2012).

## 4 Conclusion

Micellar carriers have emerged as a versatile and highly tunable platform for enhancing the efficacy and precision of PDT. By leveraging a wide variety of materials – including amphiphilic polymers, peptides, and responsive block copolymers – micelles offer improved solubility, stability, and tumor-targeted delivery of hydrophobic photosensitizers. In addition, by proper selection of the carrier material and the mode of photosensitizer loading, it is possible to favor intersystem crossing, thereby enhancing singlet oxygen quantum yields. Furthermore, such design strategies can modulate the type of photochemical reactions, potentially improving the efficacy of PDT even under hypoxic conditions.

The incorporation of stimulus-responsive mechanisms enables controlled activation of therapeutic agents within the tumor microenvironment, thereby minimizing off-target effects and systemic toxicity. These advances address several longstanding challenges in classical PDT, including poor tissue selectivity, limited penetration depth, and non-specific PS distribution. However, despite these advantages, the full therapeutic potential of micellar systems remains constrained by the inherent limitations of one-photon excitation, particularly when treating deep-seated tumors. Future developments that combine micellar carriers with strategies such as two-photon excitation, NIR-activatable systems, and multimodal imaging-guided therapies hold great promise for advancing PDT towards more effective, safe, and clinically translatable cancer treatments.

## CRediT authorship contribution statement

**Alžběta Turnovská:** Writing – review & editing, Writing – original draft, Visualization, Conceptualization. **Tomáš Etrych:** Writing – original draft, Supervision, Resources, Conceptualization.

## Declaration of competing interest

The authors declare that they have no known competing financial interests or personal relationships that could have appeared to influence the work reported in this paper.

## Data Availability

No data was used for the research described in the article.

## Footnotes

1 The Φ_Δ_ values were normalized to 0.2 % PpIX-conjugated micelles. (Ding et al., 2011a)

2 Calculated using equation $K=\left[{\mathrm{Pheo}}_{\mathrm{free}}\right]\left[\mathrm{Polymer}\right]/\left[{\mathrm{Pheo}}_{\mathrm{NP}}\right]$ based on ${\mathrm{Pheo}}_{\mathrm{NP}}⇆{\mathrm{Pheo}}_{\mathrm{free}}+\mathrm{NP}$, where NP means nanoparticle.
